# Identification of TSSK1 and TSSK2 as Novel Targets for Male Contraception

**DOI:** 10.3390/biom15040601

**Published:** 2025-04-18

**Authors:** Saman Nayyab, Marıá Gracia Gervasi, Darya A. Tourzani, Yeva Shamailova, Hiroki Akizawa, Mahboubeh Taghavi, Wei Cui, Rafael Fissore, Ana Maria Salicioni, Gunda I. Georg, Elizabeth Snyder, Pablo E. Visconti

**Affiliations:** 1 Department of Veterinary and Animal Sciences, University of Massachusetts-Amherst, Amherst, MA 01003, USA; saman.nayyab@rutgers.edu (S.N.); rfissore@umass.edu (R.F.); salicion@umass.edu (A.M.S.); 2 Molecular and Cellular Biology Program, University of Massachusetts, Amherst, MA 01003, USA; 3 Department of Animal Science, University of Connecticut, Storrs, CT 06269, USA; 4 Department of Animal Science, Rutgers University, New Brunswick, NJ 08901, USAelizabeth.snyder@rutgers.edu (E.S.); 5 Department of Cell and Developmental Biology, Perelman School of Medicine, University of Pennsylvania, Philadelphia, PA 19104, USA; 6 Animal Models Core Facility, Institute for Applied Life Sciences (IALS), University of Massachusetts-Amherst, Amherst, MA 01003, USA; wcui@umass.edu; 7 Department of Medicinal Chemistry, Institute for Therapeutics Discovery and Development, University of Minnesota, Minneapolis, MN 55455, USA; georg@umn.edu

**Keywords:** testis-specific kinases, TSSK1, TSSK2, spermiogenesis, sterility, KO, male contraception

## Abstract

The testis-specific serine kinases (TSSKs) are post-meiotically expressed in testicular germ cells. Their testis-specific expression, together with their putative role in phosphorylation pathways, suggests that TSSKs have relevant roles in spermiogenesis, sperm function, or both. Independent *Tssk3* and *Tssk6* knockout mice, as well as the double *Tssk1*/*Tssk2* KO males, are sterile. However, the double KO results are silent regarding the individual roles of TSSK1 and TSSK2. The aim of this study was to develop independent mutant mouse models of *Tssk1* and *Tssk2*, using CRISPR/Cas9, to evaluate their independent roles in reproduction. Male heterozygous pups were used to establish one *Tssk1* and two independent *Tssk2* mutant lines. Natural mating mutant *Tssk1* and *Tssk2* homozygous males but not females were found to be sterile. Additionally, homozygous males have lower sperm numbers and decreased motility, and were infertile in vitro. Anti-TSSK2 antibodies were validated against *Tssk2* mutants and used in Western blot and immunofluorescence experiments. TSSK2 is localized to the sperm head; importantly, it is present in the testes and sperm from *Tssk1* mutant mice, confirming individual mutation. Our results indicate that both TSSK1 and TSSK2 are individually essential for male reproduction and support both kinases as suitable nonhormonal male contraceptive targets.

## 1. Introduction

After undergoing meiosis, haploid round spermatids are differentiated into morphologically mature spermatozoa in a process known as spermiogenesis. During spermiogenesis, sperm undergo a series of molecular and morphological changes that include reorganization of the chromatin through the exchange of histones for protamine, formation of the flagellum, formation of the acrosome from the Golgi apparatus, degradation of proteins not needed for sperm function, and translation of spermatid and sperm-specific proteins. Among the post-meiotically expressed proteins, members of the testis-specific serine kinase (TSSK) family have been shown to be essential for spermiogenesis. The TSSK family is composed of 6 members (TSSK1 to -6), with TSSK5 being a pseudogene in humans and a putative nonfunctional protein in mice [1,2]. The other members, TSSK1, -2, -3, -4, and -6, are exclusively expressed in the testes during spermiogenesis [1,3,4,5,6] and remain in mature sperm after their transit in the epididymis [1], as reviewed in [7].

The important roles in sperm production and function of the diverse TSSK family members is highlighted by the subfertile or infertile male phenotype of studied knockout (KO) animal models. Male mice lacking TSSK4 are subfertile, as KO animals were shown to produce half the number of the pups as their wild-type (WT) littermates [8]. This subfertility phenotype was attributed to severe defects in sperm tail morphology with a concomitant decrease in sperm motility [8]. TSSK6 male (but not female) KOs are sterile in vivo and infertile in vitro [9,10]. The sterility is due to a 50% reduction in sperm count and to reduced sperm motility, as well as to a high incidence rate (about 90%) of sperm head morphology defects in comparison to their WT littermates [10]. Later, our group showed that sperm from TSSK6 KO animals are also unable to fertilize in vitro, even when the zona pellucida of the eggs is removed, indicating problems in sperm–egg fusion [10]. More recently, our group used CRISPR/CAS9 to generate three independent TSSK3 KO lines. In the three lines, *Tssk3*^−/−^ male mice were sterile [11]. Problems in the *Tssk3*^−/−^ male mice were mapped to spermiogenesis [11]. The three lines of TSSK3 KO displayed a drastically reduced number of sperm combined with severe sperm head morphology defects in comparison to their heterozygous littermates [11]. Consistent with our results, Nozawa and collaborators confirmed the same phenotype in an independent TSSK3 KO model [12]. Interestingly, TSSK3 seems to be essential for the phosphorylation of proteins related to male fertility [12].

Contrary to Tssk3, Tssk6, and Tssk4, three members of the TSSK family that have been independently knocked-out, two different groups eliminated Tssk1 and Tssk2 together, generating double-KO models [13,14]. The sequence similarity of TSSK1 and TSSK2, together with the close evolutionary origin [7], led investigators to assume redundancy in the function of these kinases. However, only the generation of independent null mutant models would test the possible TSSK1 and TSSK2 divergence in protein function. This would be of great interest to understand TSSK1 and TSSK2’s autonomous function and to position these kinases as targets for the development of a male contraceptive independently from each other. In this work, the main goal was to use CRISPR/Cas9 technology to generate individual KOs for either Tssk1 or Tssk2 to evaluate the extent to which these two kinases can compensate for each other. Our results indicate that both TSSK1 and TSSK2 have a role in spermiogenesis that leads to problems in sperm morphology and function. TSSK1 and TSSK2 mutant lines were used with commercial antibodies against these proteins via Western blot and immunofluorescence. Anti-TSSK2 antibodies detected a band at the right molecular weight (MW ~40 KDa) in wild-type and heterozygous TSSK2 testis and sperm extracts but not in homozygous mutant lines. Therefore, both TSSK2 mutant lines were considered complete knockouts and named throughout the manuscript Tssk2^−/−^ or TSSK2 KOs. On the other hand, anti-TSSK1 antibodies were not validated; consequently, it is not clear whether the TSSK1 protein is completely gone or if the Crispr/Cas9-induced truncation represents a functional knockout without complete TSSK1 loss. Consequently, throughout this manuscript, we refer to the TSSK1 mutant line as Tssk1^M/M^ or simply as TSSK1 mutants. The similar sterile phenotypes of the two single KO models indicates that although these kinases have similar functions, there is no functional compensation between TSSK1 and TSSK2, as both are required for proper sperm function.

## 2. Materials and Methods

### 2.1. Materials

All materials including reagents, antibodies, and hormones are listed in Supplementary Table S1.

### 2.2. Generation of Tssk1 Mutant and Tssk2 Knockout Lines by CRISPR/Cas9

Tssk1 and Tssk2 KO alleles were engineered at the Animal Models Core Facility of the Institute for Applied Life Sciences (IALS) following the protocol approved by the Institutional Animal Care and Use Committee (IACUC) at the University of Massachusetts Amherst. Briefly, adult female mice (8–10 weeks old) were superovulated with 7.5 IU of pregnant mare serum gonadotropin (PMSG), followed 48 h later by 7.5 IU of human chorionic gonadotropin (hCG), then paired with males of the same strain. At 20 h post-hCG injection, the females were euthanized for zygote collection. A volume of 5–10 pL containing gene-specific single-guide RNA (2 ng/µL) targeting Tssk1 (GCTGCCGTCCTCAAGCGACG) or Tssk2 (GGCAGATTTGACTTTTGCGT) and Cas9 mRNA (3.3 ng/µL) was microinjected into the cytoplasm of zygotes. The microinjected zygotes were cultured in KSOM medium at 37 °C in a humidified atmosphere of 5% CO_2_/5% O_2_ balanced with N_2_. After three days of in vitro culture, early blastocysts (E3.5) were transferred into CD-1 pseudo-pregnant female recipients (E2.5) using Nonsurgical Embryo Transfer. Pseudo-pregnancy was achieved by mating the CD-1 females with CD-1 vasectomized males 2.5 days before the embryo transfers. Only females with a visible vaginal plug were used as embryo recipients. The offspring were genotyped via PCR using gene-specific primers. All PCR amplicons were TA-cloned and verified by Sanger sequencing (Psomagen Inc., Cambridge, MA, USA) to identify founders carrying frameshift mutations. The founders were initially backcrossed with wild-type mice of the same strain to minimize potential off-target effects, followed by the intercrossing of heterozygous offspring to generate homozygous KO mice. The Tssk1 KO allele was generated and maintained on the C57BL/6J (JAX) background, while the Tssk2 KO alleles were generated and maintained on the B6D2F1 (C57BL/6J × DBA/2J, JAX) background, as hybrid females exhibit an improved superovulation response and higher mating success, facilitating more efficient KO generation.

### 2.3. Genotyping and Sanger Sequencing

Genomic DNA was extracted from mice ear biopsies using DNA lysis buffer (50 mM Tris, 1 mM EDTA, 0.5% Tween 20) with incubation at 55 °C for 30 min. The samples were centrifuged for 5 min at full speed followed by supernatant (DNA) collection. Specific custom Tssk1 and Tssk2 primers were synthesized using Integrated DNA Technology. The following primers were used: Tssk1 F: ′5-GTGTGGCAGGGATGTAGAGG-3′; Tssk1 R: 5′-TCTTGCGATCGATGATCTTG-3′; Tssk2 F: 5′-GTGTGGGGAGGGGATGTAG-3′; Tssk2 R: 5′-TCCACAAAGTCAGTGGGTGT-3′. Conventional PCR was conducted using Taq polymerase. To distinguish wild-type, heterozygous, or homozygous mutants, PCR amplicons were analyzed on a 3.4% agarose gel running at 105 mAmps. TSSK1 wild-types were identified as a single band at 192 bp, heterozygous were identified as double bands at 192 bp and 184 bp, and homozygous mutants were identified as a single band at 184 bp. TSSK2 line 1 wild-types were identified as a single band at 210 bp, heterozygous were identified as two bands at 210 bp and 200 bp, and knockouts identified as a single band at 200 bp. TSSK2 line 2 wild-types were identified as a single band at 210 bp, heterozygous were identified as two bands at 210 bp and 205 bp, and knockouts were identified as a single band at 205 bp. DNA bands were excised and purified from agarose gel using a QIAquick PCR and Gel Cleanup Kit, as per the kit’s instructions. The DNA concentration and purity were determined using a Nanodrop Spectrophotometer (BioDrop, Cambridge, UK). The samples were prepared and sent to Azenta (Plainfield, NJ, USA) for sequencing, as instructed by the company.

### 2.4. In Vivo Fertility Test

Two-month or older males of either *Tssk1* or *Tssk2* wild-type (^+/+^), heterozygous (^+/*m*^ or ^+/−^) and mutant (*^m^*^/*m*^ or ^−/−^) genotypes were mated with two wild-type C57BL/6J females in individual cages and their vaginal plugs were checked the following mornings. The plugged females were housed separately from the males and monitored for pregnancy and pups. The sexually active males were given one day of rest before housing them with additional females. Biological replicates were generated by using at least 3 different mice of each genotype. The pregnancy percentage was calculated as the number of pregnancies/number of plugs obtained multiplied by 100; the average percentage of pregnancies was determined for each genotype. The same test was conducted using female *Tssk1* and *Tssk2* mutant mice mated with C57BL/6J wild-type males.

### 2.5. Testes and Epididymis to Body Weight Analysis

The animal handling and euthanasia procedures were performed in accordance with the Animal Care and Use Committee (IACUC) guidelines of UMass-Amherst (protocol #3418). After the cervical dislocation of adult mice of each genotype of Tssk1 and Tssk2, the whole animal body was weighed on a scale, followed by weighing both the left and right dissected testicles or epididymis; all values are given in grams. The body to testis or body to epididymis weight ratio was calculated by the average of the left and right testes or epididymides over the body weight for each genotyped mouse.

### 2.6. Testis Fixing and Periodic Acid Solution (PAS) Staining

Whole testes from ≥2-month-old WT and mutant *Tssk1* and *Tssk2* line 1 male mice were collected and punctured 4–12 times with a 22 gauge needle before fixing in 4% paraformaldehyde in phosphate-buffered saline (PBS) overnight at 4 °C while rocking. After fixation, the tissues were rinsed twice with PBS and then washed 3 times with 1x PBS for 10 min each while rocking. Then, the tissues were washed and dehydrated with an alcohol gradient, cleared with xylene, and embedded in paraffin. The embedded samples were cut using a microtome at 4 microns and dried overnight on charged glass slides. The slides were deparaffinized in xylene and slowly rehydrated in a series of ethanol washes (100%, 95%, 80%, 70%, 50%) with tap or deionized (DI) water. The slides were PAS-stained following the manufacturer’s instructions. Briefly, the slides were immersed in PAS for 10 min and rinsed twice with deionized water before immersion in Schiff’s reagent for 20 min followed by a 5 min wash in running tap water. The samples were counterstained in Gill No. 3 hematoxylin solution for 4 min followed by a 2 min tap water rinse and 2 rinses in DI water. The samples were then dehydrated with alcohol and xylene and mounted with Permount. For the analysis, a minimum of three technical and biological replicates were performed.

### 2.7. Animal Usage, Sperm Collection, and Sperm Count

Following cervical dislocation, mature sperm were collected from epididymal cauda from ≥2-month-old male mice with 3–5 excisions, allowing the sperm to swim out of the tissue for 10 min in 1 mL modified noncapacitating Toyoda–Yokoyama–Hosi (mTYH) medium at 37 °C for 10 min. This medium consists of the following (concentrations are given in parenthesis): NaCl (119.3 mM), KCl (4.7 mM), CaCl_2_ × 2H_2_O (1.71 mM), KH_2_PO_4_ (1.2 mM), MgSO_4_ × 7H_2_O (1.2 mM), glucose (5.56 mM), Na pyruvate (0.51 mM), HEPES (20 mM), and gentamicin (10 mg/mL). After 10 min, the tissues were removed and the sperm samples were ready for usage. To establish sperm numbers and concentrations, 1:100 dilutions of the original sperm suspensions were made in H_2_O and counted in a hemocytometer. For sperm capacitation, the TYH medium was supplemented with 15 mM HCO_3_^−^ and 5 mg/mL of bovine serum albumin (BSA).

### 2.8. Sperm Morphology Analysis

Sperm cells were collected from each genotyped mouse. After a 10 min swim-out step in mTYH media, the cells were fixed with 1 mL of 4% paraformaldehyde (PFA) for 10 min at room temperature on a rocker. After 2 washes with PBS, the cells were resuspended in 0.5–1.0 mL PBS, depending on the cell concentration. Next, 50 μL of cells was added to poly-l-lysine #1.5 Corning coverslips and air-dried. The coverslips were mounted on Global Diamond White Glass microscope slides with Vectashield and sealed with nail polish. The slides were imaged on an inverted microscope (Eclipse TE300; Nikon, Melville, NY, USA) with a 40× objective (NA 1.49; Nikon).

### 2.9. Scanning Electron Microscopy

After sperm collection, the cells were fixed with 2.5% *v*/*v* glutaraldehyde in 0.5 M sodium cacodylate pH 7.2 for 1 h at room temperature. The fixed cells were then washed and resuspended with 0.1 M cacodylate at pH 7.2. The samples were imaged at the Core Electron Microscopy Facility at University of Massachusetts Chan Medical School with an FEI/ThermoFisher (Waltham, MA, USA) Quanta 200 MK II Field Emission SEM.

### 2.10. Computer-Assisted Sperm Analysis (CASA) Measurements

The sperm suspensions (30 μL; 2 × 10^6^ sperm/mL) were loaded into a pre-warmed 4-chamber slide (depth 100 µm) and placed on a microscope stage at 37 °C. The sperm motility was examined using the CEROS computer-assisted sperm analysis (CASA) system (Hamilton Thorne Research, Beverly, MA, USA). The default settings include the following parameters: frames acquired: 90; frame rate: 60 Hz; minimum cell size: 4 pixels; static head size: 0.13–2.43; static head intensity: 0.10–1.52; static head elongation: 5–100. At least four microscopy fields corresponding to a minimum of 200 sperm were analyzed for each treatment in each experiment. The percentage of hyperactive sperm was calculated based on the total motile sperm, considering hyperactive sperm as those having the following parameters: curvilinear velocity (VCL) > 271.00 μm/s, linearity (LIN) < 50.00%, and amplitude of lateral head (ALH) > 3.50 µm.

### 2.11. Sperm Anti-Phospho PKA Substrates (pPKAs) and Anti Phosphotyrosine (pY) Western Blots

After sperm collection, the cells were exposed to capacitating mTYH for either 0 or 60 min at 37 °C. The cells were then centrifuged at 12.6 rpm for 2 min and washed with 0.6 mL of ice-cold PBS. The cells were then treated with 10 μL of 5× nonreducing Laemmli sample buffer [15] boiled for 4 min at 100 °C, and centrifuged at max speed for 5 min, and then the supernatant was collected. After the addition of a 5% final concentration of β-mercapthoethanol, the protein lysates were boiled for an additional 5 min and loaded onto an 8% SDS–polyacrylamide gel for electrophoresis and electro-transferred to an Immobilon-P polyvinylidene difluoride (PVDF) membrane. The membrane was blocked with 5% fat-free milk in T-TBS for 1 h at room temperature and then treated with 1:10,000 polyclonal anti-pPKAs overnight at 4 °C in the dark. The following day, the PVDF membrane was washed 3× and incubated with IgG anti-rabbit (1:10,000) in 1% fat-free milk in T-TBS for 1 h at room temperature. After washing 3× in T-TBS, the membrane was treated with an ECL Prime Plus kit, and a signal for the pPKAs was detected. The PVDF membranes used for the pPKAs were then stripped at 55 °C for 20 min in 2% SDS, 0.74% β-mercaptoethanol, and 62.5 mM Tris at pH 6.5, followed by six washes for 5 min each in T-TBS prior to membrane blocking with 20% gelatin in T-PBS for 1 h at room temperature. They were then treated with anti-pY (1:10,000) in T-PBS and washed for 1 h at room temperature. Secondary IgG anti-mouse (1:10,000) was added, and after 30 min the membrane was washed 3× with T-PBS and the pY signal was detected using ECL Regular. A quantitative analysis was performed using ImageJ 1.47 V software (National Institutes of Health, Bethesda, MD, USA). The regions of interest (ROIs) used for quantification are indicated by a # on the left of the respective Western blot. The extent of hexokinase tyrosine phosphorylation does not change during capacitation [16] and was used as the loading control. Therefore, the optical density of the bands was normalized to tyrosine-phosphorylated hexokinase.

### 2.12. In Vitro Fertilization (IVF)

Mouse cumulus–oocyte complexes (COCs) were collected from 8–10-week-old superovulated CD1 females (Charles River Laboratories, Wilmington, MA, USA). For superovulation, the females were first injected with 7.5–10 IU of pregnant mare serum gonadotropin (PMSG) (Lee BioSolutions, cat # 493-10, Maryland Heights, MO, USA) followed by 7.5–10 IU of human chorionic gonadotrophin (hCG) (Sigma, cat # CG5, St. Louis, MO, USA) 48 h later. Thirteen hours post-hCG injection, the COCs were collected in TL-HEPES medium (NaCl (114 mM), KCl (3.22 mM), NaHCO_3_ (2.02 mM), NaH_2_PO_4_ (0.348 mM), HEPES (10.1 mM), lactic acid (sodium salt) (10 mM), CaCl_2_.2H_2_O (1.71 mM), MgCl_2_.7H_2_O (1.2 mM), gentamicin (10 mg/mL))_,_ with the addition of bovine serum albumin (5 mg/mL) on the day of experiment, washed in mTYH (see above for composition), and placed in the insemination droplet. *Tssk1*^+/+^, *Tssk1*^+/*M*^, *Tssk1^M^*^/*M*^, *Tssk2*^+/+^, *Tssk2*^+/−^, and *Tssk2*^−/−^ from line 1 and line 2 male mice sperm were collected as described previously, and approximately 100,000 sperm were added to a 90 µL insemination droplet. After 4 h, the oocytes were washed in mTYH and allowed to culture for 20 h. The following day, fertilization was determined via the visualization of cleavage into a 2-cell stage embryo.

### 2.13. Intracytoplasmic Sperm Injection (ICSI)

ICSI was performed as previously reported by [17] using the described setup and micromanipulators (Narishige, Japan). Sperm from *Tssk1*^+/+^, *Tssk1*^+/*M*^, *Tssk1^M^*^/*M*^, *Tssk2*^+/+^, *Tssk2*^+/−^, and *Tssk2*^−/−^ from line 1 and line 2 male mice (≥2 months old) were collected from the cauda epididymis in mTYH and washed three times in the same media; then, heads were separated from tails via sonication (XL2020; Heat Systems Inc., Phoenix, AZ, USA) for 5 sec at 4 °C. The sperm lysate was washed in mTYH and diluted with 12% polyvinylpyrrolidone (PVP) to a final PVP concentration of 6%. A piezo micropipette driving unit was used to deliver the sperm into the ooplasm (Primetech, Ibaraki, Japan); a few piezo-pulses were applied to puncture the eggs’ plasma membrane following penetration of the zona pellucida. After ICSI, the eggs were cultured in KSOM to evaluate the 2-cell development at 36.5 °C in a humidified atmosphere containing 5% CO_2_.

### 2.14. TSSK2 Protein Detection via Western Blots

#### 2.14.1. Testes

Testis protein extracts were obtained [16] from *Tssk1*^+/+^, *Tssk1*^+/*M*^, *Tssk1^M^*^/*M*^, *Tssk2*^+/+^, *Tssk2*^+/−^, and *Tssk2*^−/−^ from line 1 and line 2 male mice. Briefly, both the left and right testes were dissected from ≥2-month-old mice and the tunica albuginea was removed in Dulbecco’s modified Eagle’s medium (DMEM). The seminiferous tubes were cut into small fragments and transferred into a disaggregation cartridge. The fragments were manually processed in the cartridge for 5 min and passed through 40 μm cell strainers twice. The samples were then processed again manually in the disaggregation cartridge for 5 min. Once germ cells from the testes were obtained, the protein was extracted using the same method as for the sperm cells.

#### 2.14.2. Sperm

After mature sperm collection from TSSK2 wild-type, het, and KO mice, the cells were washed with ice-cold PBS. The cell pellets were treated with 1x Laemmli buffer [15], 1x protease inhibitor, and 2 mM of dithiothreitol and incubated on ice for 30 min, with vortexing every 5 min. The cells were then sonicated for 3 sections (10 sec pulse and 30 sec pause) on ice. The cells were centrifuged at 15,000× *g* for 20 min at 4 °C and 95 μL of supernatant was collected. After the addition of 5% β-mercapthoethanol, the samples were boiled for 5 min at 100 °C, then 30 μg of protein was loaded onto an 8% SDS–polyacrylamide gel for electrophoresis and electro-transferred to a PVDF membrane. The membrane was blocked with 5% milk in T-PBS for 1 h at room temperature and then treated with anti-TSSK2 (clone 1E12) at a concentration of 2 μg/mL overnight at 4 °C in the dark. The following day, the membrane was washed 3× with T-PBS and incubated with secondary IgG anti-mouse (1:10,000) in 1% milk in T-PBS for one hour at room temperature. The membrane was then washed 3× with T-PBS and the TSSK2 signal was detected with an ECL Prime Plus kit. Equal loading was determined with blot stripping and reprobing was performed with monoclonal α anti-tubulin (E7).

### 2.15. TSSK2 Immunofluorescence

#### 2.15.1. Sperm

TSSK2 wild-type, het, or KO epididymal caudal sperm were collected from ≥2-month-old mice and fixed in 1 mL 4% PFA in 1x PBS for 10 min at room temperature. After fixing, the cells were centrifuged at 150× *g* for 5 min and washed 2x with 1 mL PBS. The cells were resuspended in 0.1 mL–1 mL PBS, depending on the sperm concentration. Then, 50 μL of cells was added to poly-l-lysine #1.5 Corning coverslips and air dried at room temperature. The cells were permeabilized with 0.5% *v*/*v* Triton X-100 in PBS for 5 min at room temperature and then washed 3× with 0.01% Tween-PBS (T-PBS) for 5 min each time. The cells were blocked with 10% BSA in T-PBS for 1 h at room temperature. After the hour blocking, anti-TSSK2 (clone 1E12) was added at a 20 μg/mL concentration in 1% BSA in T-PBS and incubated at 4 °C overnight in the dark. The next day, the coverslips were washed 3× with T-PBS and treated with anti-mouse IgG AlexaFluoro 488 (1:1000), peanut agglutinin (1:100), and Hoechst (1:100) for one hour at room temperature. The cells were then washed 5× with 1x T-PBS for 5 min in the dark at room temperature and mounted on Global Diamond White Glass microscope slides with Vectashield. The slides were imaged on an inverted microscope (Eclipse TE300; Nikon) with epifluorescence and DIC images were taken with a 60x PlanApo/DIC objective (NA 1.49; Nikon).

#### 2.15.2. Testes

The testes were dissected from adult mice and fixed overnight in 4% PFA. The tissue was rinsed in PBS and dehydrated in increasing concentrations of ethanol before embedding in paraffin wax and sectioning to 4 μm. Antigen retrieval was performed as previously described [18]. Briefly, the slides were boiled in Tris-EDTA (10 mM Tris-HCl, 1 mM EDTA, and 0.05% Tween (pH 9.0)) for 30 min on low. The slides were blocked in 4% goat serum for 1 h followed by primary antibodies (anti-TSSK2 1:250; anti-SYCP3, 1:1000) overnight at room temperature. The slides were washed in 0.1% Triton PBS then incubated with goat anti-rabbit (1:1000) and goat anti-mouse (1:1000) secondary antibodies for 1 h at room temperature. The slides were washed in 0.1% Triton PBS, followed by 1x PBS, and mounted using DAPI Fluoromount-G. Once dry, they were stored at 4 °C with light protection until imaging. The slides were visualized on a custom-built microscope (Zeiss, Jena, Germany) with fluorescent and bright-field capabilities. The provided images are representative of three or more biological samples. The signal intensity was matched across slides by matching the background (interstitial) signal intensity. The developmental stages were determined according to the parameters set forth by [19,20].

### 2.16. Statistics

Statistical analyses were performed using Prism 8.4.3 software (by GraphPad). Data are expressed as the mean ± SEM. All data were verified to accomplish the parametric assumptions of homogeneity of variances and normality. Variables were compared via a one-way analysis of variance, and the statistical significance between groups was determined using Tukey’s post-hoc test. Statistical analyses for optical densitometries were performed using two-tailed *t*-tests ranked for parametric data and a Wilcoxon matched pair ranked test for nonparametric data. Significance was considered as * *p* < 0.05, ** *p* < 0.01, *** *p* < 0.0005, or **** *p* < 0.0001.

## 3. Results

### 3.1. Generation of Individual Tssk1 and Tssk2 Knockout Mice Using CRISPR/Cas9

**Figure 1:**
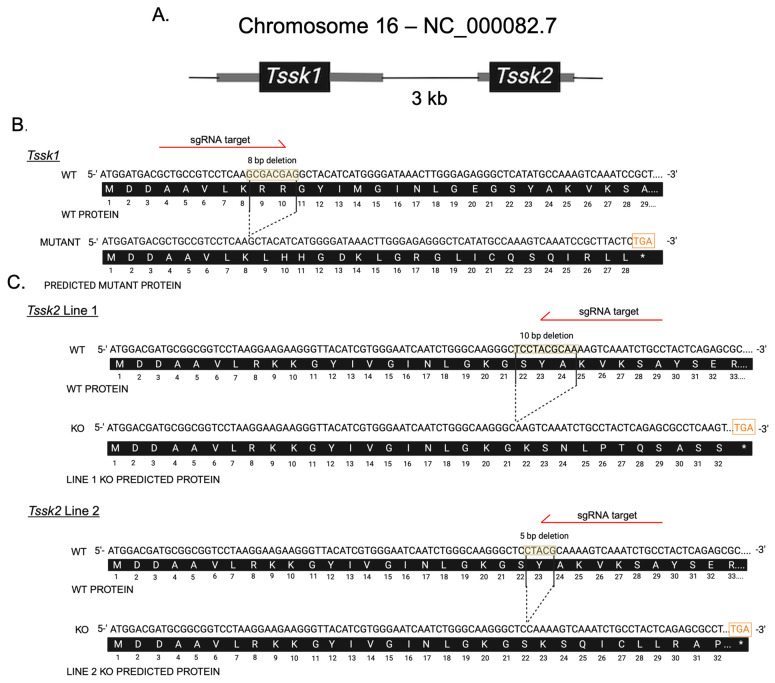
Generation of Tssk1 and Tssk2 mutant mouse models. *Tssk1* mutant and *Tssk2* mutant mouse models generated using CRISPR/Cas9 technology. (**A**) Schematic representation of the *Tssk1* and *Tssk2* locus in mouse chromosome 16. The 5′ and 3′ UTR regions are depicted in gray, ORF is depicted in black. (**B**,**C**) Fragment of the genomic sequences of the reference WT gene with red arrows depicting sgRNA target sites for (**B**) mutated *Tssk1* and (**C**) *Tssk2* lines. For each line, deleted bases are indicated within dotted lines. Premature stop codon is indicated by an orange box in the DNA sequence and by the asterisk in the predicted protein sequence. Wild-type and predicted protein sequences are indicated below each line (white letters over black background).

In the mouse genome, both *Tssk1* and *Tssk2* are intronless genes located on chromosome 16, only 3 kilobase pairs apart (Figure 1A). To generate individual *Tssk1* and *Tssk2* mutant mouse models, we synthesized sgRNAs targeting the initial sequence immediately after the respective first methionine. These sgRNAs were injected in one-cell embryos together with CRISPR/Cas9, and the resulting embryos were cultured in vitro up to the blastocyst stage and then transferred into pseudo-pregnant females. In fertility tests, those males with disruptions in both alleles of the respective *Tssk1* or *Tssk2* sequences from the F0 generation that produced plugs were unable to induce pregnancies in CD1 females (Supplemental Table S1). To establish stable colonies, we used heterozygous males and generated one *Tssk1* mutant line with an 8 bp deletion on the *Tssk1* ORF (Figure 1B) and two *Tssk2* KO lines consisting of a 10 bp deletion (line 1) and a 5 bp deletion (line 2) on the *Tssk2* gene (Figure 1C). The three deletion mutants originated nucleotide frameshifts that generated early stop codons in the respective *Tssk* gene (Figure 1B,C). The results from PCR genotype sequencing confirmed truncated *Tssk1* in *Tssk1^M^*^/*M*^ animals and truncated *Tssk2* in *Tssk2*^−/−^ animals (Supplement Figure S1A,B). Conversely, in the *Tssk1* mutant line, the *Tssk2* gene was not affected, and in the *Tssk2* mutant lines, the *Tssk1* wild-type gene was complete (Supplement Figure S1C).

### 3.2. Tssk1^M/M^ and Tssk2^−/−^ Males but Not Females Are Sterile

**Figure 2:**
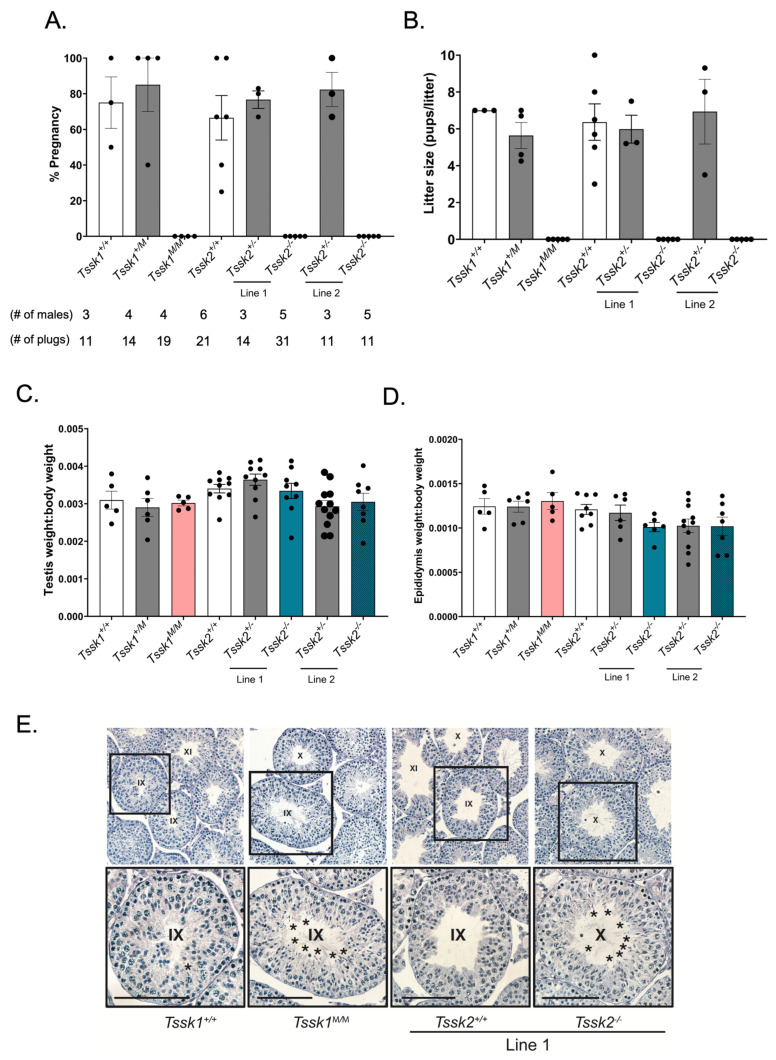
Analysis of male fertility in vivo. (**A**) Male mice from the respective genotype were mated with female mice as described in the Methods. The percentage of pregnancy rates of *Tssk1*^+/+^, *Tssk1*^+/*M*^, and *Tssk1^M^*^/*M*^ and both lines of *Tssk2*^+/+^, *Tssk2*^+/−^, and *Tssk2*^−/−^ animals were calculated as the number of pregnancies/number of plugs obtained multiplied by 100. (**B**) Litter size indicated by average number of pups per litter per male of *Tssk1*^+/+^, *Tssk1*^+/*M*^, and *Tssk1^M^*^/*M*^ and both lines of *Tssk2*^+/+^, *Tssk2*^+/−^, and *Tssk2*^−/−^ animals. (**C**) Ratio of testis weight vs. body weight collected from *Tssk1*^+/+^, *Tssk1*^+/*M*^, and *Tssk1^M^*^/*M*^ and both lines of *Tssk2*^+/+^, *Tssk2*^+/−^, and *Tssk2*^−/−^ animals. (**D**) Ratio of adult epididymis weight vs. body weight collected from *Tssk1*^+/+^, *Tssk1*^+/*M*^, and *Tssk1^M^*^/*M*^ and both lines of *Tssk2*^+/+^, *Tssk2*^+/−^, and *Tssk2*^−/−^ animals. Statistical comparison of corresponding wild-type samples with *Tssk1*^+/*M*^ and *Tssk1^M^*^/*M*^ and both lines of *Tssk2*^+/−^ and *Tssk2*^−/−^ using a one-way ANOVA, followed by Tukey’s test. (**E**) Adult testis histological evaluation of *Tssk1*^+/+^, *Tssk1*^+/*M*^ and *Tssk1^M^*^/*M*^ and both lines of *Tssk2*^+/+^, *Tssk2*^+/−^, and *Tssk2*^−/−^. Stages indicated by Roman numerals. Asterisks indicate retained spermatids. Upper row: 10× magnification; lower row: approximately 20× magnification, scale bar = 100 um.

To determine the fertility of mutant males, all three genotypes of *Tssk1* and *Tssk2* were mated independently with C57BL/6J WT females over the span of two months (see Methods Section). The females exhibited no difference in the numbers of plugs among all male genotypes, indicating no change in mating behavior in the mutants. However, while the WT and heterozygous males of each line has similar pregnancy rates and litter size numbers, all mutant males presented a sterility phenotype (Figure 2A,B). On the other hand, the *Tssk1* and *Tssk2* mutant females were fertile. No differences in testis or epididymis size or weight were found in the homozygous mutant lines when compared to either wild-type or heterozygous samples (Figure 2C,D).

In order to gain insight into the potential driver of male sterility in *Tssk* mutants, we examined the testicular morphology and germ cell development in *Tssk1* and *Tssk2* line 1 mutants (Figure 2E). This analysis demonstrated a normal tubule structure and relatively normal germ cell development, with the mutant testes containing the same complement of germ cells as observed in the wild-type. However, the detailed analysis revealed the mutant testes often had mature spermatozoa associated with tubules that in wild-type testes contain only immature spermatids (stages IX through XI), suggesting defective spermiation. The wild-type testes rarely displayed mature spermatozoa in stage IX, and if so, they were few in number (~1 per tubule). Further, no retained spermatozoa were observed in the wild-type testes beyond stage IX. In contrast, the stage IX tubules of *Tssk1* mutants nearly always contained large numbers of retained spermatozoa. The *Tssk2* line 1 mutants showed an even more severe defect, with many retained spermatozoa through to stage X and in some cases into stage XI.

**Figure 3:**
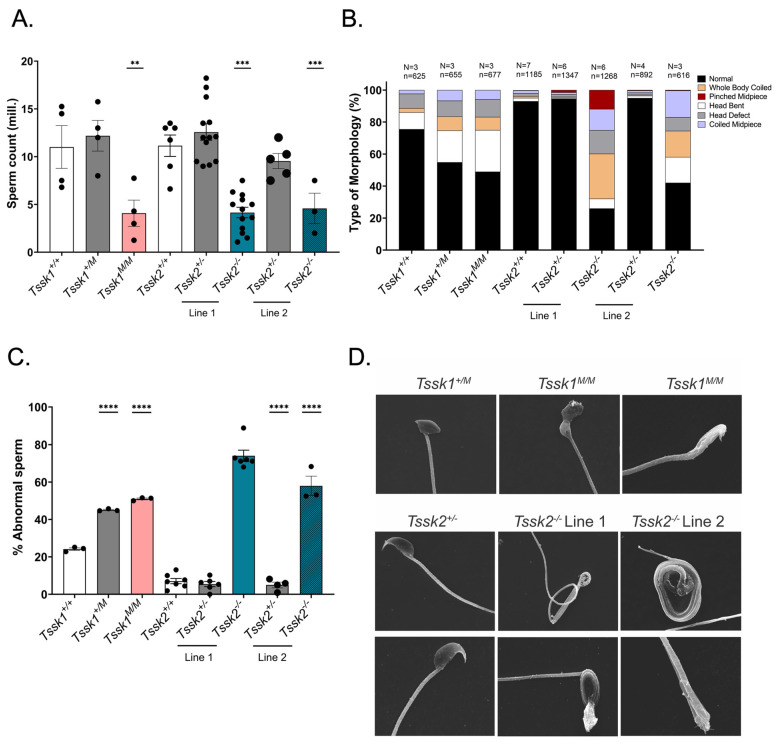
Phenotypic analyses of *Tssk1* and both lines of *Tssk2*. (**A**) Numbers of sperm recovered from the cauda epididymis of *Tssk1*^+/+^, *Tssk1*^+/*M*^, and *Tssk1^M^*^/*M*^ and both lines of *Tssk2*^+/+^, *Tssk2*^+/−^, and *Tssk2*^−/−^ animals. Data are presented as the mean + SEM; *p* < 0.01 (**), or 0.0001 (***). (**B**) Quantification of sperm morphological types of *Tssk1*^+/+^, *Tssk1*^+/*M*^, and *Tssk1^M^*^/*M*^ and both lines of *Tssk2*^+/+^, *Tssk2*^+/−^, and *Tssk2*^−/−^ animals. Sperm morphology types were categorized as normal, whole body coiled, pinched midpiece, head bent, head defects, and coiled midpiece. N = number of independent replicates; n = total number of sperm counted. (**C**) Percentage of abnormal sperm morphology calculated as number of defective sperm/total number of sperm counted multiplied by 100. Statistical comparison of corresponding wild-type samples with *Tssk1*^+/*M*^ and *Tssk1^M^*^/*M*^ and both lines of *Tssk2*^+/−^ and *Tssk2*^−/−^ sperm using one-way ANOVA, followed by Tukey’s test, *p* < 0.0001 (****). (**D**) Scanning electron microscopy images of *Tssk1*^+/*M*^ (minor head defect) and *Tssk1^M^*^/*M*^ (major head defects) and both lines of *Tssk2*^+/−^ (normal heads) and *Tssk2*^−/−^ (whole body coiled and severe head defects) sperm show the severity of morphological defects in mutant sperm; 5000× magnification.

Despite the overall normal testis and epididymis weights and structures, the number of epididymal sperm in all homozygous mutants was significantly reduced (Figure 3A). Further, both the *Tssk1* homozygous and heterozygous mutants exhibited a higher number of sperm abnormalities than wild-type samples (Figure 3B,C and Supplementary Figure S2A). In the case of the *Tssk2* mutant lines, only the homozygous *Tssk2*^−/−^ presented abnormalities. We observed different types of abnormalities, including head defects and complete tail coiling (see Supplementary Figure S2A for pictures of the respective defects), and calculated their distributions (Figure 3B), as well as the accumulated defects (Figure 3C). Scanning electron microscopy was used to confirm these abnormalities in detail (Figure 3D and Supplementary Figure S2B).

**Figure 4:**
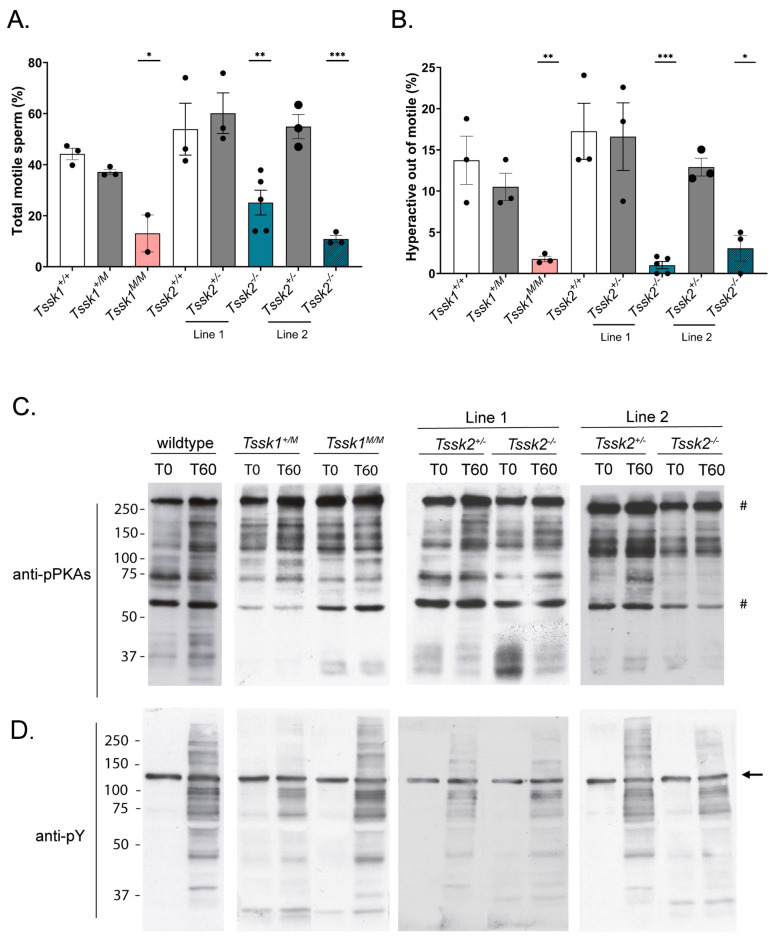
Sperm motility and phosphorylation pathways. (**A**) Percentage of motile sperm and (**B**) percentage of hyperactive motility at 60 min in capacitating media were analyzed using a computer-assisted sperm analysis (CASA), as explained in the Methods. Statistical comparison of corresponding wild-type samples with *Tssk1*^+/*M*^ and *Tssk1^M^*^/*M*^ and both lines of *Tssk2*^+/−^ and *Tssk2*^−/−^ sperm using a one-way ANOVA, followed by Tukey’s test at *p* < 0.05 (*), *p* < 0.01 (**), *p* < 0.0005 (***). (**C**,**D**) Representative Western blots of wild-type, *Tssk1*^+/*M*^, *Tssk1^M^*^/*M*^ and both lines of *Tssk2*^+/+^, *Tssk2*^+/−^, and *Tssk2*^−/−^ animal sperm protein after 0 or 60 min in capacitating media probed with (**C**) anti-pPKAs and (**D**) anti-pY antibody. N = 3. The region between the two # signs indicates the region used for pPKA quantification. The arrow indicates tyrosine-phosphorylated hexokinase [21] and the region below the arrow was used for pY quantification.

Next, we investigated how the sperm function was affected in each of the *Tssk* mutants. The percentage of motile sperm was significantly reduced in both *Tssk2*^−/−^ lines and *Tssk1^M^*^/*M*^ (Figure 4A). Importantly, the percentage of hyperactive sperm was close to zero (Figure 4B) in mice homozygous for either *Tssk1^M^*^/*M*^ and *Tssk2*^−/−^. These defects in motility parameters were not due to problems in capacitation-associated phosphorylation pathways (Figure 4C and Figure S3). We also tested the ability of the sperm from the *Tssk1^M^*^/*M*^ and *Tssk2*^−/−^ mouse models to fertilize in vitro (Table 1). The fertilization rate was zero for *Tssk1^M^*^/*M*^ and almost zero for *Tssk2*^−/−^ line 1. The heterozygotes *Tssk2*^+/−^ and *Tssk1*^+/*M*^ fertilization rates were slightly reduced compared to their wild-type siblings; however, this reduction was not statistically significant. On the other hand, when intracellular sperm injections (ICSIs) were conducted, similar cleavage rates were found in the sperm from all genotypes tested (Table 2).

### 3.3. TSSK2 Localization in Sperm and Testes

**Figure 5:**
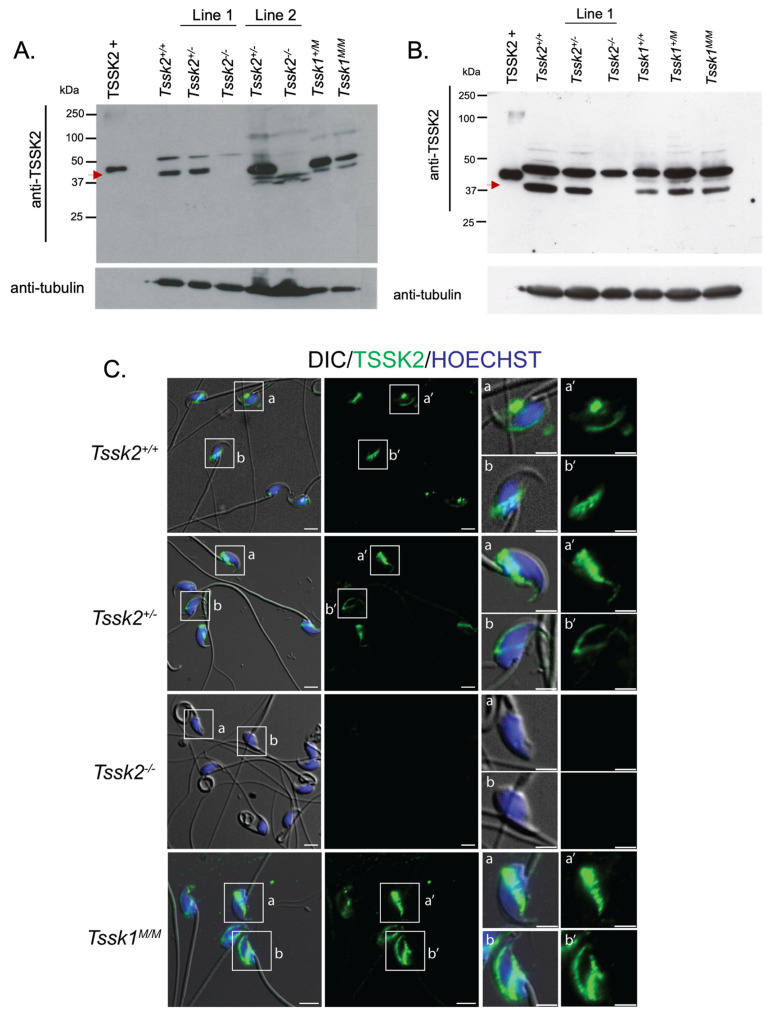
Validation and use of anti-TSSK2 antibodies. (**A**) Western blot of testis extracts from *Tssk2*^+/+^; both lines of *Tssk2*^+/−^, *Tssk2*^−/−^, and *Tssk1*^+/*M*^; and *Tssk1^M^*^/*M*^. (**B**) Western blot of sperm extracts from *Tssk2*^+/+^ and line 1 of *Tssk2*^+/−^; *Tssk2*^−/−^; and *Tssk1*^+/+^, *Tssk1*^+/*M*^ and *Tssk1^M^*^/*M*^ animals. A specific band detected by an anti-TSSK2 antibody is indicated by an arrow at ~40 KDa. Anti-tubulin was used as the loading control. (**C**) Representative images show differential interference contrast (DIC) and epifluorescence microscopy of caudal sperm indicating TSSK2 localization from *Tssk2*^+/+^, both lines of *Tssk2*^+/−^ and *Tssk2*^−/−^, and *Tssk1^M^*^/*M*^. TSSK2 is shown in green and nuclear staining with Hoechst in blue. Scale bar = 20 μm; insert scale bar = 5 μm. (Figure S5).

As mentioned, we showed via the PCR analysis that the *Tssk1* gene is complete in the *Tssk2*^−/−^ background and that the *Tssk2* gene is complete in the *Tssk1^M^*^/*M*^ background. However, these results are silent regarding the presence of the respective protein products. To further evaluate the respective proteins, we used commercially available antibodies against TSSK1 and TSSK2. As mentioned in the introduction, from these antibodies, we were only able to validate antibodies against TSSK2. In both *Tssk2* lines, the anti-TSSK2 antibody detected a band of the expected 40 KDa of MW in wild-type and heterozygous testes (Figure 5A) and sperm (Figure 5B) extracts. That band was undetectable in the KOs. The 40 KDa band was also observed in lysates from testes and sperm from *Tssk1^M^*^/*M*^ mice, confirming that the *Tssk1* mutation did not impact TSSK2 production. Therefore, for the localization studies, we only purchased the anti-TSSK2 antibody.

Using immunofluorescence, we detected TSSK2 in the sperm head (Figure 5C and Supplemental Figure S4) of heterozygous and homozygous *Tssk1* mutant sperm. The signal was not observed in sperm from *Tssk2*^−/−^ mice, further confirming the specificity of the anti-TSSK2 antibody. Specifically, TSSK2 localized to the anterior head and to the postacrosomal region, in a localization process similar to the one observed for TSSK6 [9]. Consistent with the Western blots, TSSK2 was also detected in sperm from *Tssk1^M^*^/*M*^ mice.

**Figure 6:**
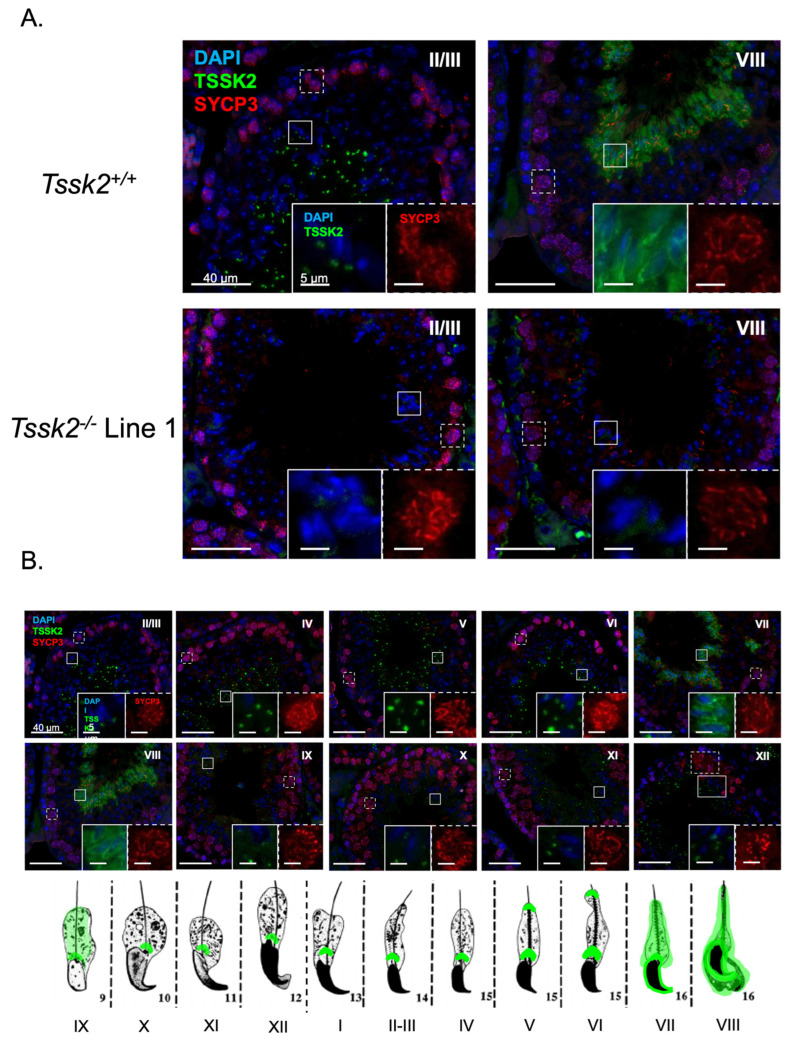
Localization of TSSK2 during spermatid development. (**A**) Immunofluorescence of TSSK2 (green) in adult testes. DAPI (blue) and SYCP3 (red) localization used to determine the stage of the cycle of the seminiferous epithelium [18]. The corresponding stage is indicated with a Roman numeral. (**B**) Immunofluorescence of TSSK2 (green) by stage. Summary of spermatid TSSK2 localization by spermatid type (numerals) correlated to stage (Roman numerals). Spermatid images derived from [20] DAPI (blue) and SYCP3 (red) co-staining for stage determination.

**Figure 7:**
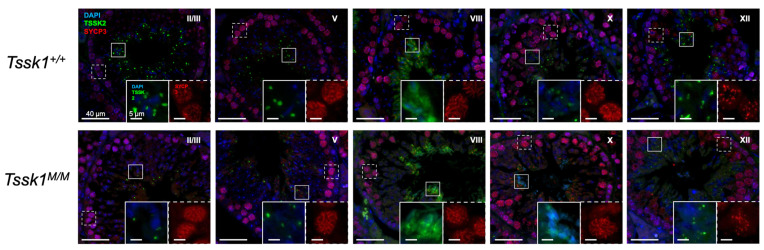
Immunofluorescence of TSSK2 (green) in *Tssk1* mutant testes, with DAPI (blue) and SYCP3 (red) localization used to determine stage of the cycle of the seminiferous epithelium [19]. The respective stage is indicated by a Roman numeral.

To better define the developmental regulation of TSSK2 during spermiogenesis, we performed immunofluorescence on adult wild-type and *Tssk2* KO (Figure 6A) testicular sections. As anticipated based on the Western blots, the *Tssk2*^−/−^ mutants showed no TSSK2 signal in either spermatids or mature testicular spermatozoa. Developmentally (Figure 6B), TSSK2 was first detected in step 9 elongating spermatids, primarily as a single focus near the base of the nucleus with an occasional weak cytoplasmic signal. The cytoplasmic signal was entirely lost by step 10. Over the course of spermatid elongation, the single TSSK2 focus resolved into two distinct foci, one near the nuclei and the other moving distal, possibly along the growing spermatid tail (Figure 6B). These findings are in general agreement with previous reports [14]. The focus-based localization remained until step 16, when the TSSK2 became generally cytoplasmic. To determine whether TSSK2 localization is impacted by the mutation of *Tssk1*, we next examined TSSK2 in the context of *Tssk1* mutants (Figure 7). Overall, the *Tssk1* mutants displayed a slight delay in focal accumulation of TSSK2, a potential reduction in TSSK2 focal intensity or size, and a moderate delay in focal dissolution. Although cell-intrinsic defects or delays resulting from *Tssk1* mutation cannot be ruled out, these results confirm that *Tssk1* mutants express TSSK2 and suggest that TSSK2 localization may be dependent on TSSK1.

## 4. Discussion

Spermatogenesis encompasses a series of events that culminate in the postmeiotic differentiation of haploid spermatids. This last step is known as spermiogenesis and involves the conversion of round spermatids into elongated spermatozoa. Spermiogenesis includes the formation of the sperm acrosome and the flagellum, redistribution of the mitochondria to the sperm tail, synthesis of new proteins, and elimination of others through the activation of degradation and secretory pathways. Importantly, spermiogenesis induces chromatin compaction through the exchange of histones for protamines. All of these changes in the spermatid architecture rely on finely controlled signaling pathways; among them, post-translational modifications, such as phosphorylation, play essential roles. The importance of phosphorylation pathways is highlighted by a growing number of protein kinases that are abundantly expressed in the testis and have been shown to be critical for spermiogenesis [7,22]. Among these kinases, the testis-specific serine kinase (TSSK) family has six members named numerically from 1 to 6. The focus of this manuscript is on TSSK1 and TSSK2.

*Tssk1* was discovered in 1994 using a degenerate primer strategy [3], and three years later, the same group identified TSSK2 using ^32^P-labeled *Tssk1* as the probe [4] to screen a mouse testis cDNA library. In the mouse, *Tssk1* and *Tssk2* are encoded in chromosome 16 and separated by only 3 kb. In humans, *TSSK2* is found in the DiGeorge syndrome minimal region in chromosome 22. This region in chromosome 22 is syntenic to the mouse chromosome 16; however, in higher primates, *TSSK1* in the syntenic chromosome 22 location is a pseudogene. Interestingly, a new *TSSK* member with high homology to the mouse *Tssk1* is found in a nonsyntenic region in chromosome 5. This gene was named *TSSK1b* and is hypothesized to have appeared in higher primates through a retro-transposition event [13]. Importantly, in humans, *TSSK1b* is also mainly expressed in the testes. At the same time of the *TSSK2* discovery, Ziemiecki’s group reported that both TSSK1 and TSSK2 phosphorylate another testis-specific protein, and they named it testis-specific kinase substrate (TSKS) [4].

Taking advantage of the proximity between the *Tssk1* and *Tssk2* genes in the mouse, two groups generated double-knockout mouse models lacking both genes using a homologous recombination in ES cells [13,14]. One of these groups was not able to generate progeny lacking both genes, and it was concluded that these knockout mice were haploinsufficient [14]; the second group was able to generate homozygous mutant mice [13]. This last group showed that the double *Tssk1*^−/−^*/Tssk2*^−/−^ mice have a male sterility phenotype without presenting defects in females. This manuscript highlighted the relevance of these kinases for reproduction; however, the work was silent regarding the individual TSSK1 and TSSK2 roles. Here, we took advantage of CRISPR/Cas9 methodology to generate mutant mice that eliminated the functional expression of either TSSK1 or TSSK2 and evaluated their phenotype. We found that individual mutations in both genes generated male sterility without affecting female reproduction. In both mutant models, the overall testis and epididymis anatomy was not altered but the number of spermatozoa and their morphology were significantly affected. Moreover, a higher percentage of the *Tssk2* mutant spermatozoa had motility problems, while both *Tssk1* and *Tssk2* homozygous mutants were not able to undergo hyperactivation. Given these phenotypic characteristics, it was not surprising to find that the sperm from these mice were not able to fertilize metaphase II-arrested eggs in vitro; interestingly, they were able to generate two-cell embryos when injected by ICSI, suggesting that chromatin condensation and decondensation were not affected.

We also used these mouse models as controls for specific antibodies. However, we were only able to validate anti-TSSK2 antibodies, which recognize specific Western blot bands in sperm and testis extracts from *Tssk2*^+/+^ and *Tssk2*^+/−^ but not in extracts from *Tssk2*^−/−^ mouse models. Therefore, follow-up experiments were only conducted with the validated anti-TSSK2 antibody. Using this antibody, we showed that TSSK2 is present in sperm and testis extracts from *Tssk1*^−/−^ mutants. This result indicated that TSSK2 is still expressed in mice lacking the functional expression of TSSK1. We also used the anti-TSSK2 antibody to localize TSSK2 in the sperm and testes. Consistent with previous results [1], TSSK2 has a post-acrosomal localization like the one observed for TSSK6 [9]. In addition to this localization, TSSK2 was found in the anterior head. In the testes, TSSK2 is observed in post-meiotic spermatids, with variable localization based on spermatid developmental state. Consistent with previous reports [13], this refined localization pattern suggests dynamic regulation of TSSK2 localization during spermatid development, specifically during spermatid elongation, and begs the question of why TSSK2 has such a distinct localization pattern. Based on the wide range of mutant sperm phenotypes, it is tempting to postulate that TSSK2 may play a dual role in regulating sperm head morphology and sperm tail formation, in particular the midpiece. These suppositions are also consistent with the localization pattern of TSSK2 in mature sperm. A second question arising from these findings is whether the two TSSK2 foci share distinct or similar protein substrates. Based on the previous localization of TSKS [13], overlapping substrates seems highly likely. Regardless, directly addressing these questions will require detailed localization, protein binding, and substrate identification studies of TSSK2.

Our results indicate that both TSSK1 and TSSK2 are essential for male reproduction. These findings add to the growing body of evidence indicating the critical role of kinases and phosphorylation in spermiogenesis. As an example, the recent work by Pasand et al. indicates that a methamphetamine-induced increase in inflammatory cell populations decreases TSSK protein expression [23]. In addition to TSSK1 and TSSK2, among the relevant spermatid kinases required for male reproduction, we can mention TSSK3 [11,12], TSSK6 [10], HIPK4 [24], and STK33 [25]. In addition, mice lacking TSSK4 are sub-fertile [8]. As previously published [7], the TSSK family is evolutionarily conserved. Recent studies in Drosophila melanogaster (D.m.) showed that the relevance of this kinase family for male reproduction is also conserved. Contrary to mammals, in D.m., only one *dTssk* gene (aka CG14305) is found; this gene has between 37 and 46% shared identity with mammalian TSSKs, with the highest homology to TSSK1 and TSSK2. The elimination of this gene from the D.m. genome using CRISPR/Cas9 resulted in a sterile fly phenotype [24]. Flies lacking the *dTssk* gene present severe spermiogenesis defects, including impairment of the histone-to-protamine transition and morphological defects. As part of this work, the authors crossed *dTssk*^−/−^ flies with transgenic flies expressing human *TSSK1B*, *TSSK2*, *TSSK3*, *TSSK4*, or *TSSK6* under the control of the endogenous *dTssk* promoter. Each of the individual human TSSKs partially restored spermiogenesis defects but none of them was able to rescue the sterility phenotype. Interestingly, in *dTssk*^−/−^ flies, *TSSK1B* (the TSSK1 human homologue) but not *TSSK2* rescued nucleus morphology, as well as defects in histone-to-protamine transition [26]. The differential ability of each human TSSK to rescue spermiogenesis defects in *dTssk*^−/−^ flies suggests that during evolution, the dTSSK function was divided between multiple TSSKs.

From a clinical perspective, a deletion of ~8-Mb in the 5q22.2q23.1 locus harboring the TSSK1B gene causes asthenoteratozoospermia [27]. The same group performed Sanger sequencing of the coding regions and exon borders of TSSK1B in a cohort of 100 male patients with unresolved infertility causes. They discovered missense mutations in 10% of the cases that were associated with sperm abnormalities, including two previously unreported TSSK1B mutations [28]. In 2013, a study found that 5 out of 372 infertile Chinese men who had oligospermia or azoospermia contained some mutation in the *Tssk4* gene. The study found 4 single nucleotide changes within the gene: 2 within the 5′ and 3′ untranslated region (UTR) and two in exon 3 [29]. These clinical reports, together with loss-of-function studies in flies and mice, including those in this paper, strongly suggest that small molecules blocking TSSK activity will induce reversible sterility in men and other species. Reproductive control is a key aspect of human health. It is estimated that about 50% of pregnancies worldwide are unintended, and this has a direct impact, primarily in women of reproductive age and society at large [30]. Currently, the burden of family planning relies on women, leaving men with limited options [31]. In recent years, the efforts to develop safe and effective male-directed contraceptives have significantly increased.

Ideally, male contraceptive targets should exclusively affect reproductive processes. Among the different strategies, hormonal approaches such as exposure to testosterone induces suppression of spermatogenesis via the inhibition of gonadotropin release [32,33]. More recently, efforts have been made to find nonhormonal compounds that target reproductive molecules. These compounds have higher safety and efficacy requirements than other small molecules targeting diseases. As is also the case for women’s contraceptives, the small molecules that block male reproduction should have negligible or no side effects and ideally should be completely reversible. The targets for nonhormonal male contraceptives should have some relevant properties: (1) they should be expressed exclusively or almost exclusively in reproductive cells and tissues, including testis, epididymis, or germ cells or spermatozoa; (2) they should be amenable for finding drugs that inhibit their function; (3) they should be essential for male reproduction. TSSKs have all of these properties; they are testis-specific proteins and are druggable, and loss-of-function models showed that they are indispensable for male reproduction without affecting female reproduction or other tissues. In this regard, the work from our group has produced recombinant TSSK2 [34] and identified potent TSSK1 and TSSK2 inhibitors having pyrrolopyrimidine and pyrimidine cores [35]. Some of these compounds have IC50s in low nanomolar concentrations; however, their selectivity is still poor. These findings warrant the use of molecular medicine strategies to achieve higher selectivity.

## 5. Conclusions

Previously, only double-KO mouse models of Tssk1 and Tssk2 genes have been evaluated [13,14]; these studies indicate that the elimination of both kinases results in a male sterility phenotype. Although our results will require confirmation in other mammalian species, the present study indicates that the genomic integrity of both Tssk1 and Tssk2, independently of each other, is essential for male reproduction in mouse models. At the protein level, a lack of validation of anti-TSSK1 antibodies prevented some additional conclusions regarding its cellular localization, as well as its presence or absence in Tssk2^−/−^ mouse models. The present work, together with other studies using loss-of-function strategies, is consistent with the hypothesis that TSSKs are essential for spermiogenesis. However, the lack of in vitro spermiogenesis models makes the elucidation of the signaling pathways in which these kinases are involved difficult. The generation of Tssk KO mice is a step forward to understanding their function. In addition, we expect that new findings and approaches will contribute significantly to the elucidation of TSSK pathways. In this respect, the recent work by Delgado et al. [36] indicates that TSSK6 promotes oncogenic behavior in colorectal cancer. Although it is likely that the roles of TSSK6 in cancer and in the testes are not the same, the use of cell culture systems will allow a testable hypothesis to be generated. Similarly, the aforementioned studies in Drosophila melanogaster warrant a more tractable experimental system to investigate TSSK signaling pathways. Finally, we expect that in addition to their translational application in male concentrations, specific small-molecule inhibitors will provide invaluable tools for basic research on TSSK pathways.

## Figures and Tables

**Table 1:** In-vitro fertilization rate.

Tssk1	Fertilization (%)	N
+/+	275/326 (84.09)	3
+/*M*	217/305 (71.78)	3
*M*/*M*	0/238 (0)	3
***Tssk2***	**Fertilization (%)**	**N**
+/+	96/105 (91.40)	2
Line 1 +/−	172/219 (78.50)	6
Line 1 −/−	3/180 (1.67)	5

**Table 2:** Intracytoplasmic sperm injection rate.

Tssk1	Fertilization (%)	N
+/+	27/39 (69.23)	3
+/*M*	44/66 (66.67)	3
*M/M*	47/68 (69.12)	3
***Tssk2***	**Fertilization (%)**	**N**
+/+	32/36 (88.89)	3
Line 1 +/−	37/46 (80.43)	3
Line 1 −/−	52/67 (77.61)	3
Line 2 +/−	22/29 (75.86)	3
Line 2 −/−	16/29 (55.17)	2

## Data Availability

The data generated or analyzed in this study can be found within the published article and its Supplementary Files.

## Supplementary Materials

The following supporting information can be downloaded at: https://www.mdpi.com/article/10.3390/biom15040601/s1, Table S1: F0 Fertility Test; Figure S1: PCR amplicon and Sanger sequencing confirm deleted region in mutant Tssk1 and Tssk2 knock out mouse models; Figure S2: Abnormal sperm morphology images; Figure S3: Quantitative analysis of optical density of pPKAs and pY blots; Figure S4: Additional immunofluorescence experiments using anti TSSK2 antibodies. Figure S5: Original Images for Western Blots.
