# Supplementary material for: Identification of TSSK1 and TSSK2 as Novel Targets for Male Contraception
Source: Biomolecules. 2025 Apr 18;15(4):601. doi: 10.3390/biom15040601 (PMC12024862; doi:10.3390/biom15040601)
Supplement: Supplementary file 1 [file biomolecules-15-00601-s001.zip › biomolecules-3481730-Figure S5.pdf]

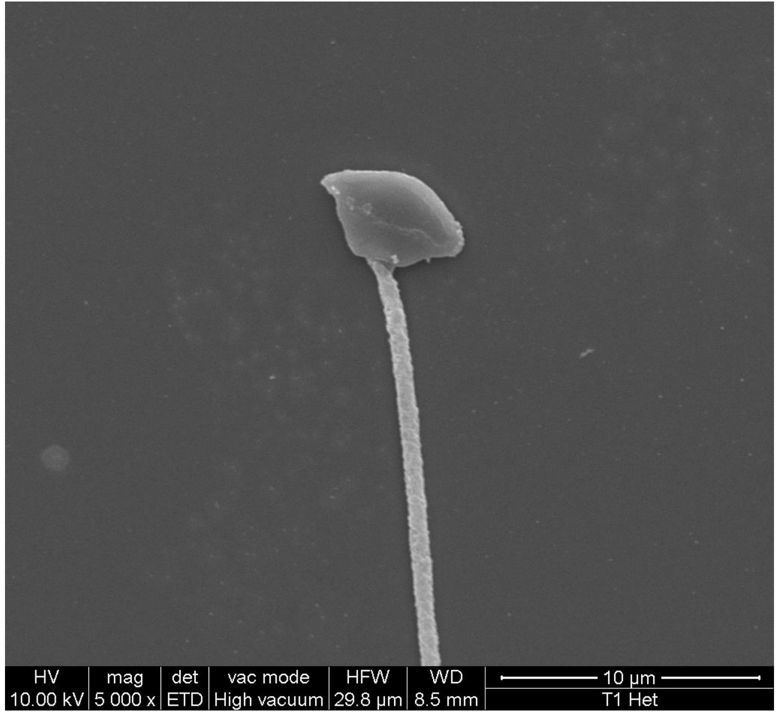

Tssk1$^{+/M}$

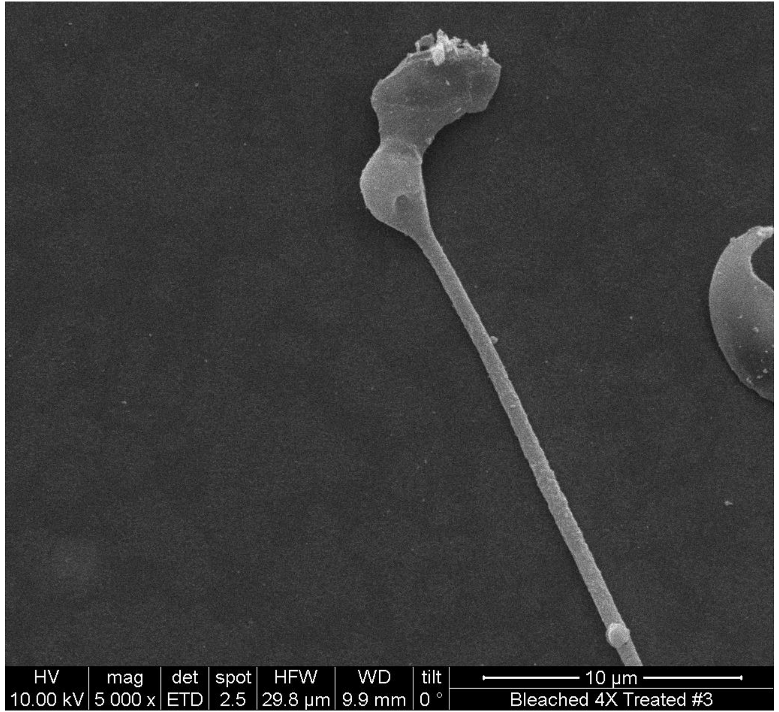

Tssk1$^{M/M}$

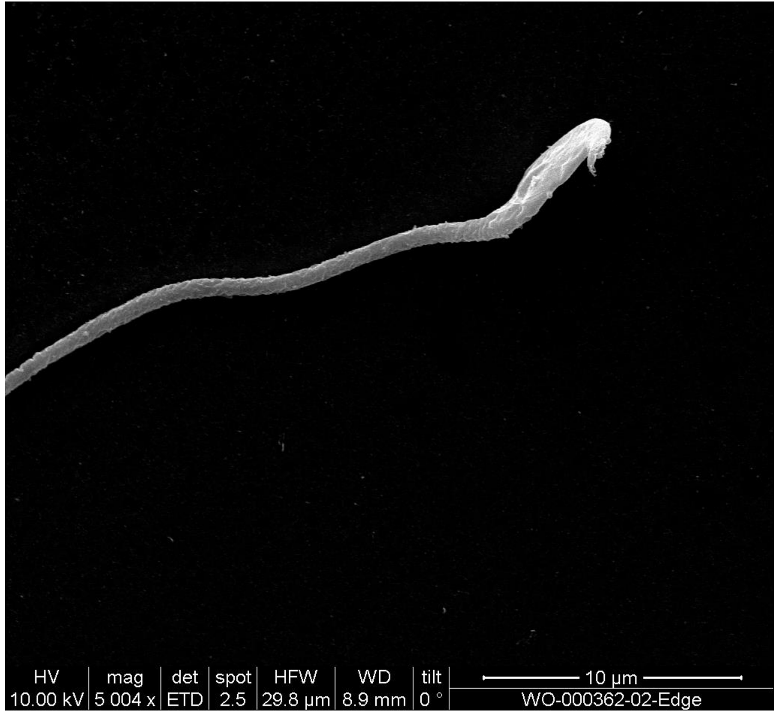

Tssk1$^{M/M}$

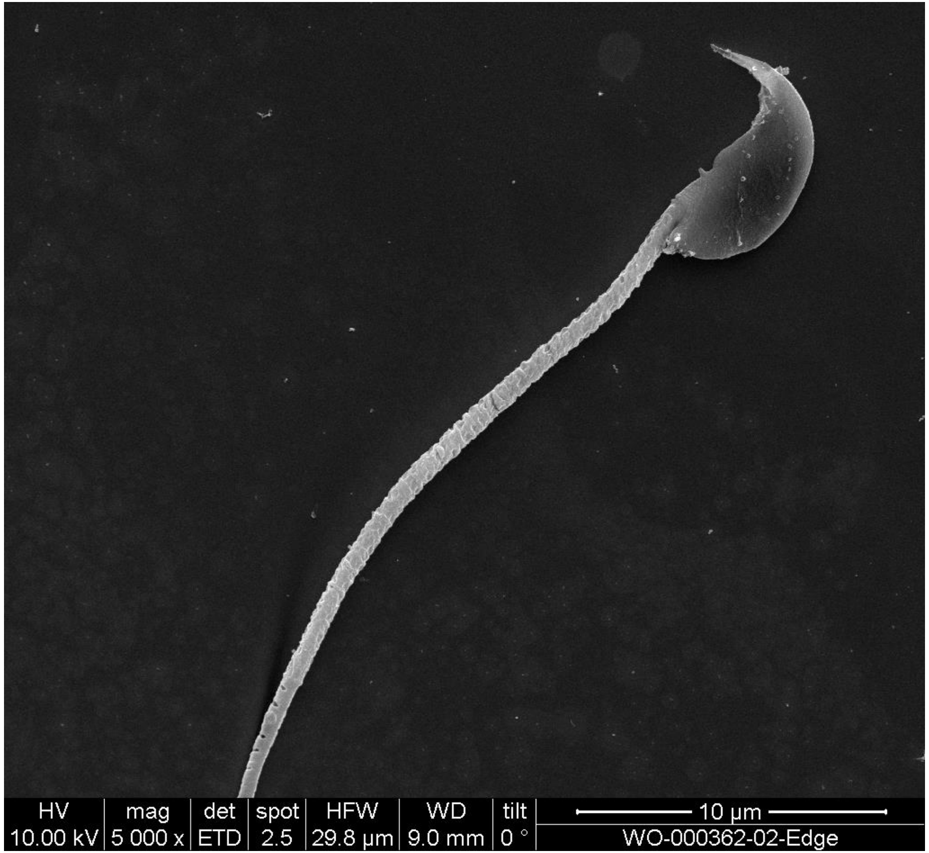
HV
10.00 kV
mag
5 000 x
det
ETD
spot
2.5
HFW
29.8 µm
WD
9.0 mm
tilt
0 °
10 µm
WO-000362-02-Edge

Tssk2$^{+/-}$

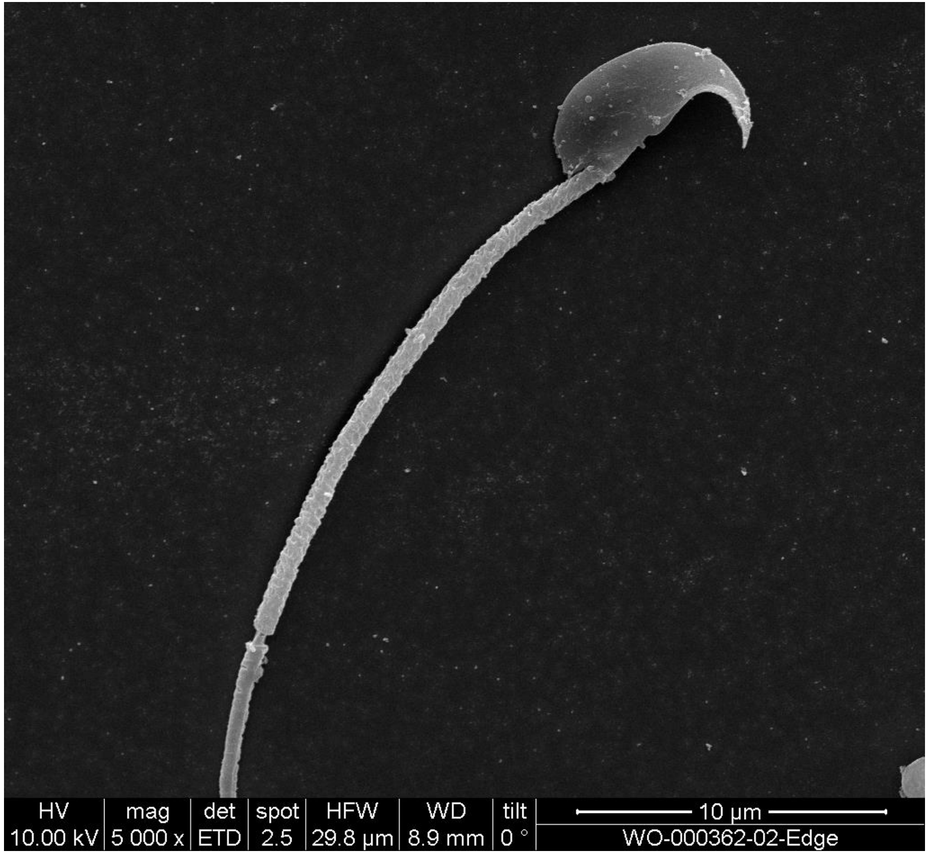
HV
10.00 kV
mag
5 000 x
det
ETD
spot
2.5
HFW
29.8 µm
WD
8.9 mm
tilt
0 °
10 µm
WO-000362-02-Edge

Tssk2$^{+/-}$

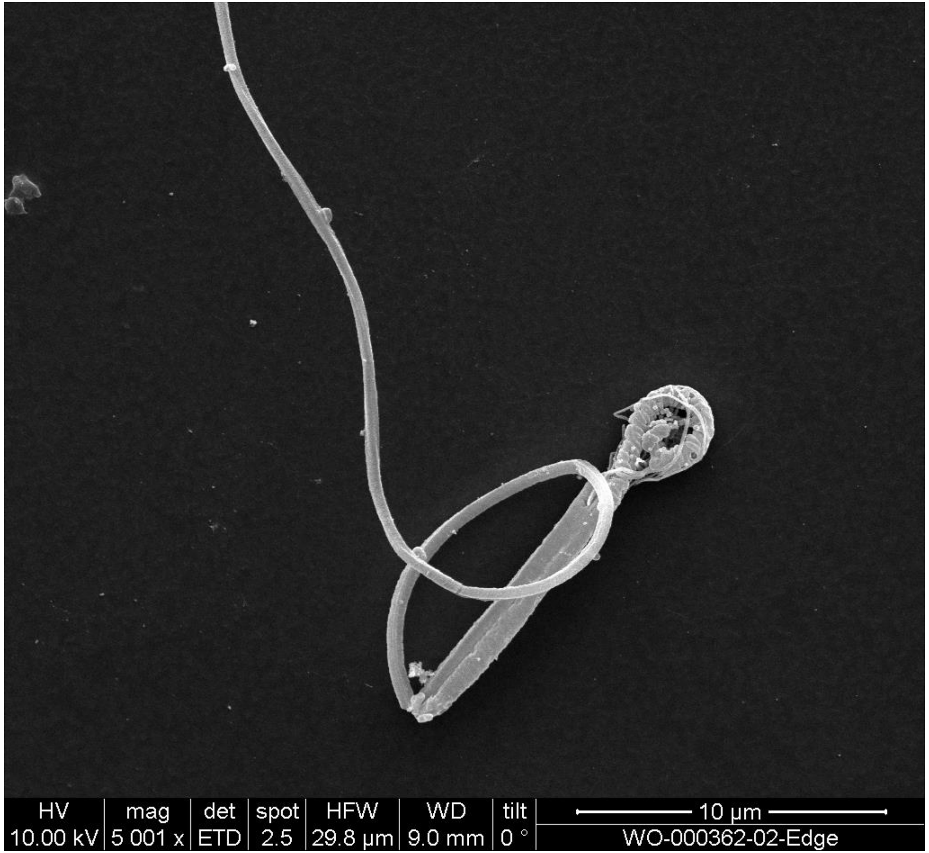

Tssk2$^{-/-}$ Line 1

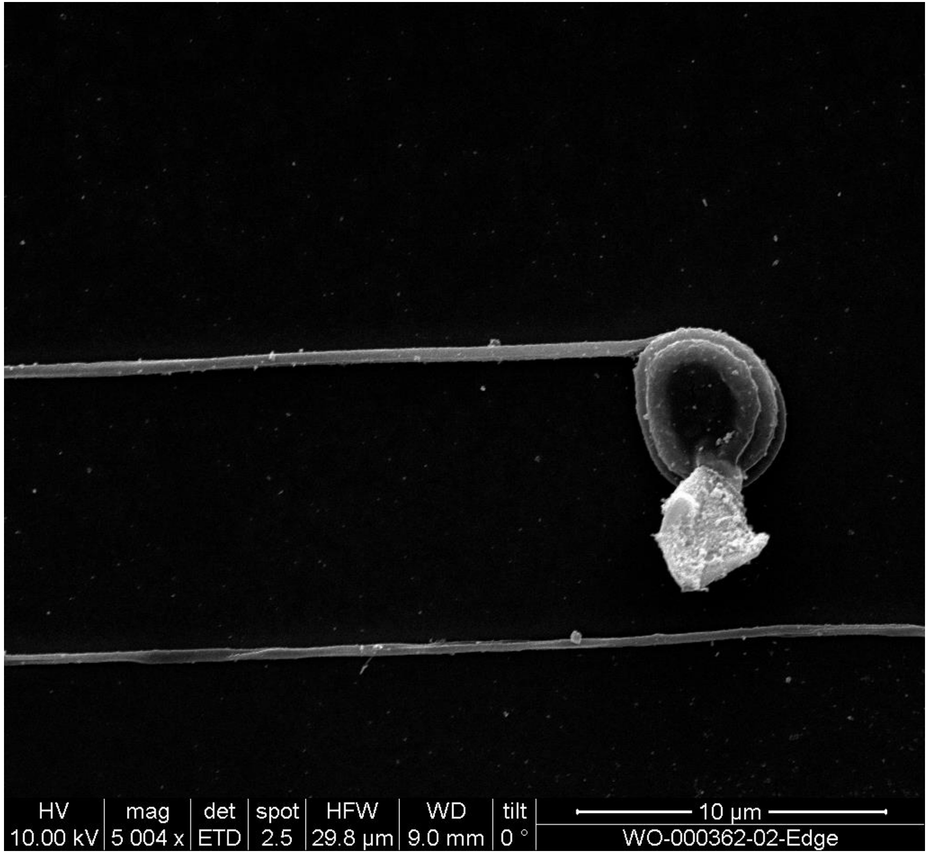

Tssk2$^{-/-}$ Line 1

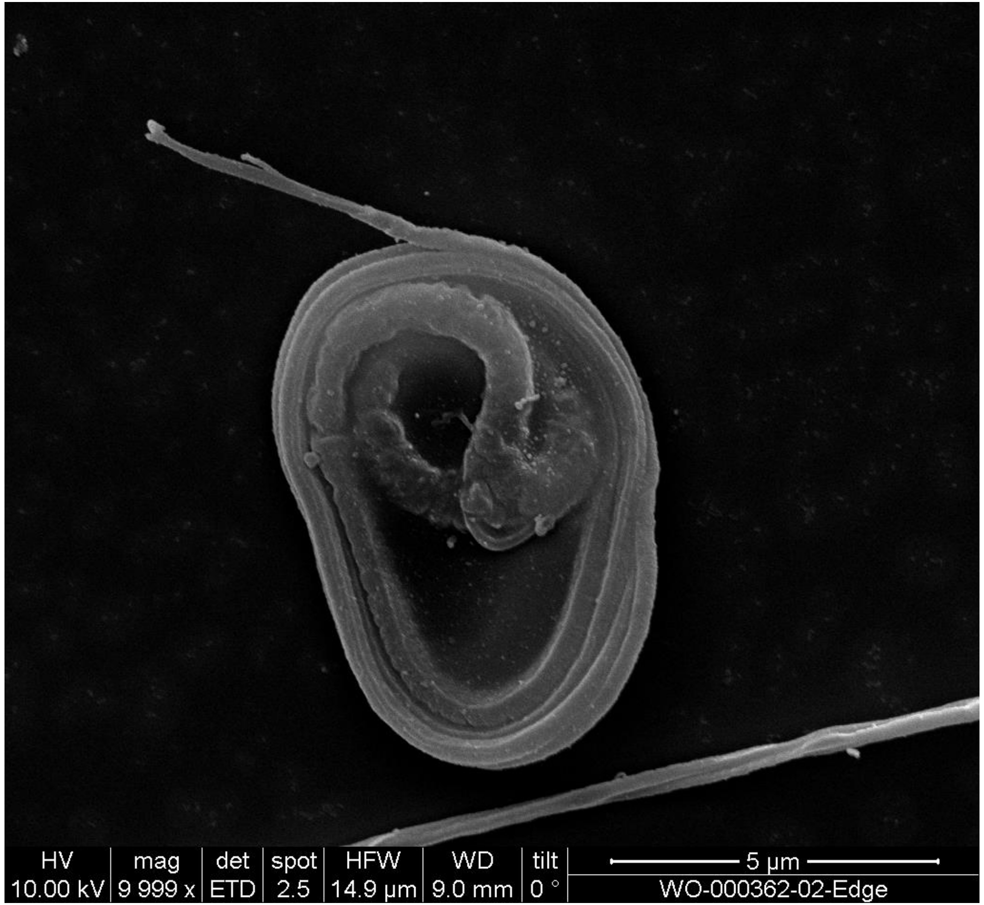

Tssk2$^{-/-}$ Line 2

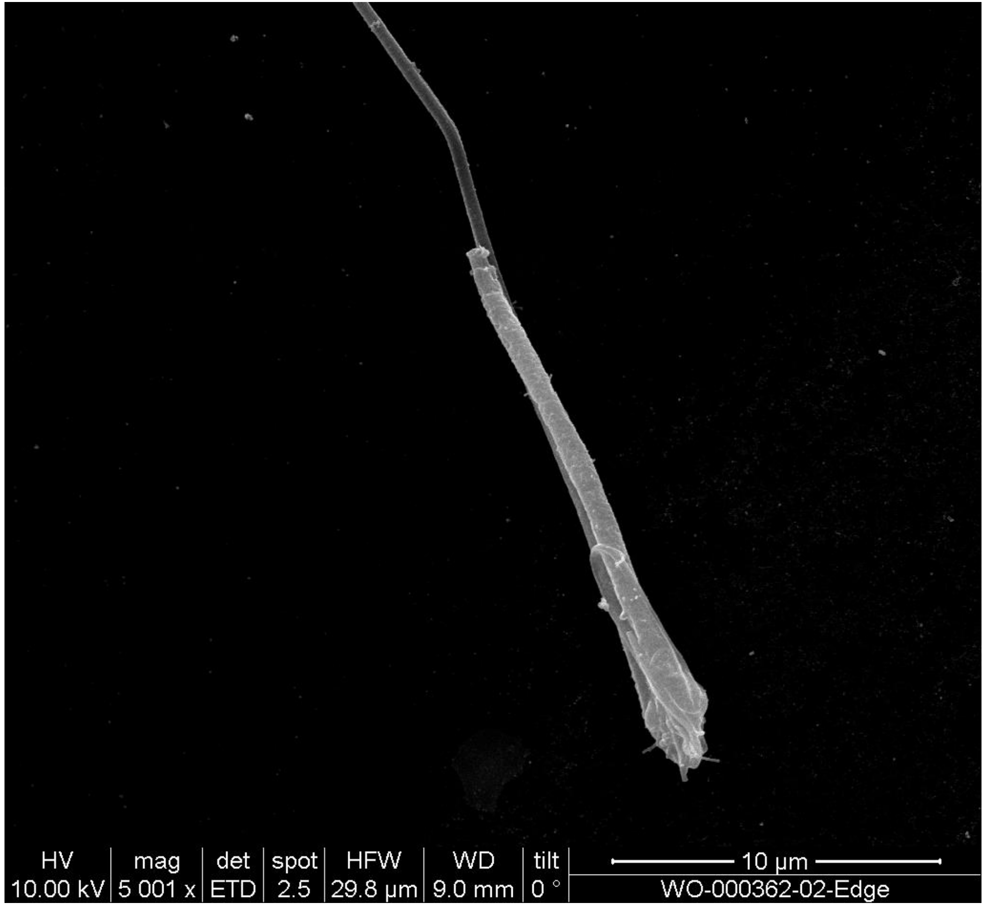

Tssk2$^{-/-}$ Line 2

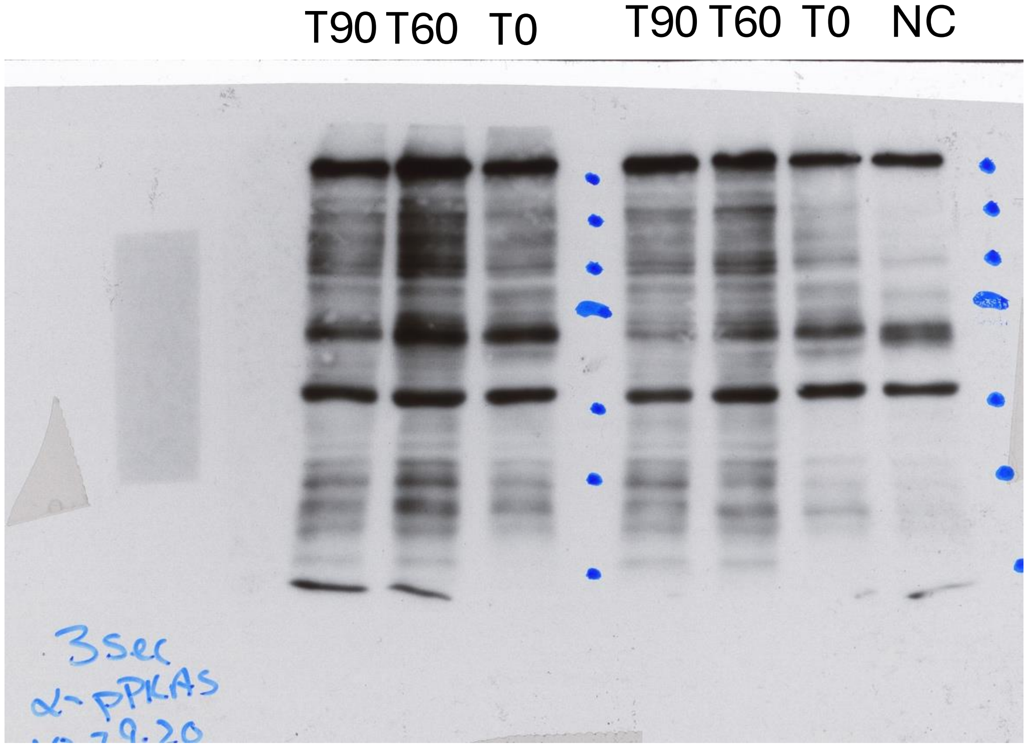

4C wildtype pPKA

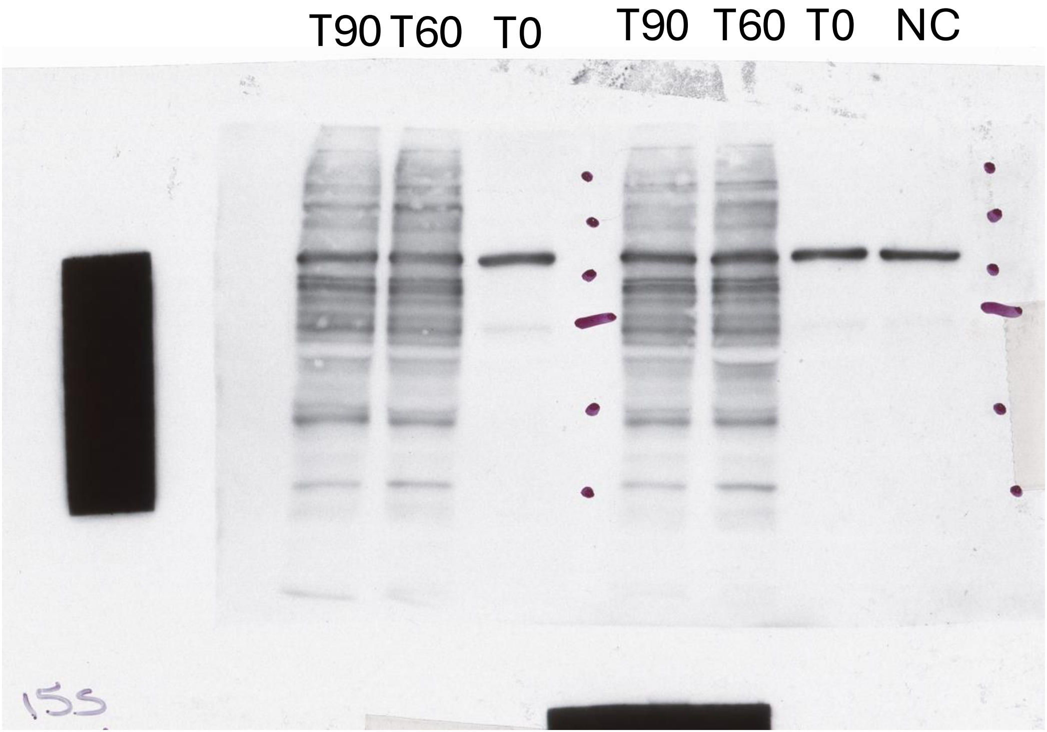

4C wildtype pY

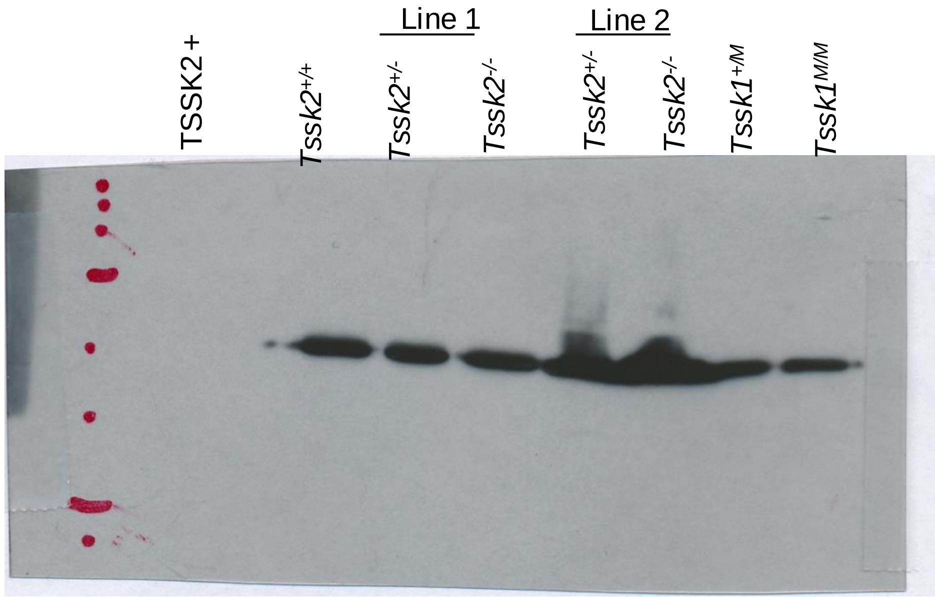

5A tubulin

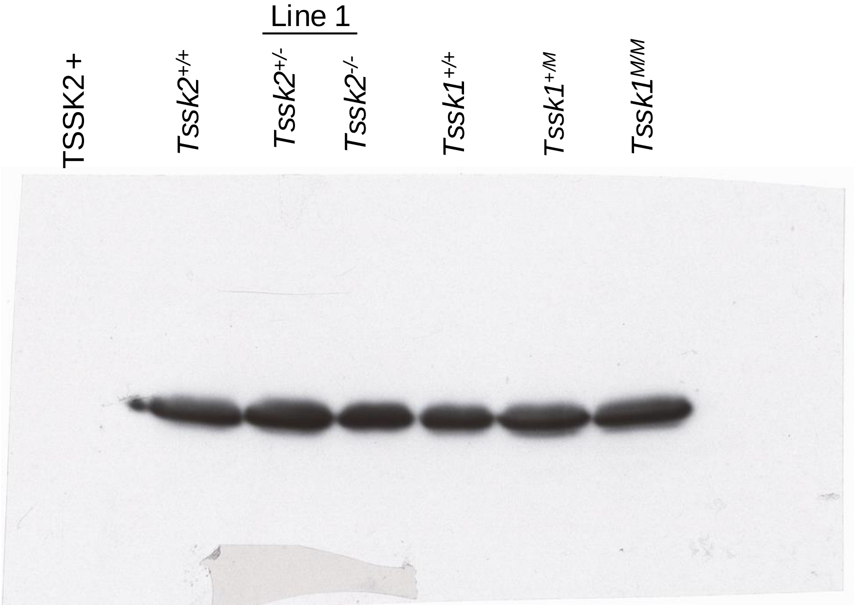

5B tubulin
