# Supplementary material for: Identification of TSSK1 and TSSK2 as Novel Targets for Male Contraception
Source: Biomolecules. 2025 Apr 18;15(4):601. doi: 10.3390/biom15040601 (PMC12024862; doi:10.3390/biom15040601)
Supplement: Supplementary file 1 [file biomolecules-15-00601-s001.zip › biomolecules-3481730-supplementary.pdf]

Supplement Table S1: F0 Fertility Test

GENOTYPE	PLUG #	PUP #
***Tssk1*$^{+/-}$**	8	22
***Tssk1*$^{M/M}$ #1**	7	0
***Tssk1*$^{M/M}$ #2**	6	0
***Tssk1*$^{M/M}$ #3**	1	0
***Tssk2*$^{+/+}$**	4	15
***Tssk2*$^{-/-}$ #1**	5	0
***Tssk2*$^{-/-}$ #2**	5	0
***Tssk2*$^{-/-}$ #3**	5	0

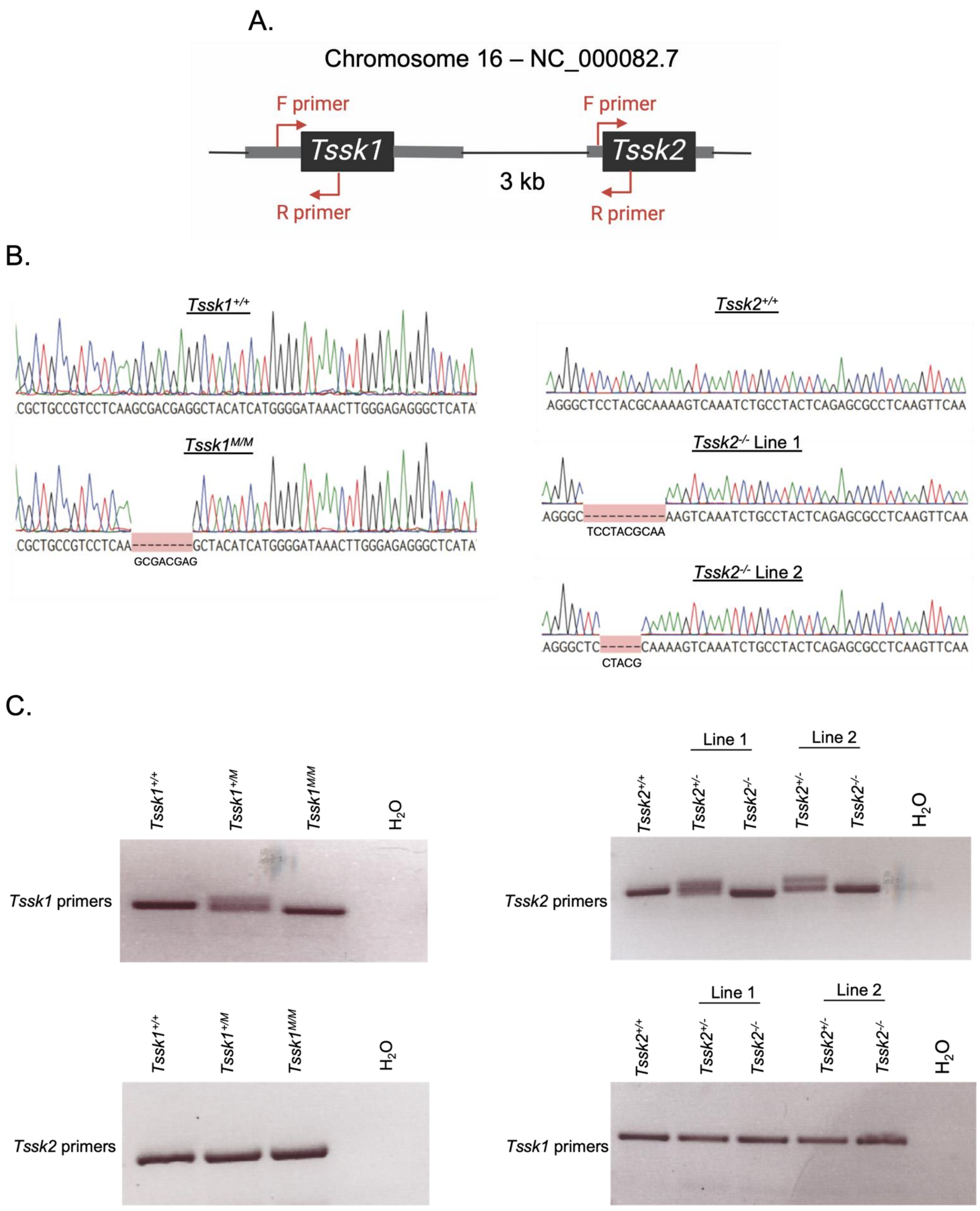

Supplement Figure S1. PCR amplicon and Sanger sequencing confirm deleted region in mutant *Tssk1* and *Tssk2* knockout mouse models. A) Schematic representation of the *Tssk1* and *Tssk2* locus in the mouse chromosome 16. The 5' and 3' UTR regions are depicted as thick gray lines, ORF is depicted in black. Red arrows indicate annealing site of forward and reverse primers used for PCR for *Tssk1* and *Tssk2*. **B)** PCR amplicon of *Tssk1* and *Tssk2* from *Tssk1*$^{+/+}$, *Tssk1*$^{+/M}$, *Tssk1*$^{M/M}$ animals (left) and from *Tssk2*$^{+/+}$, both lines of *Tssk2*$^{+/-}$, and *Tssk2*$^{-/-}$ animals (right) amplified with either *Tssk1* specific primers or *Tssk2* specific primers. **C)** Sanger sequence of *Tssk1*$^{+/+}$ and *Tssk1*$^{M/M}$ and *Tssk2*$^{+/+}$, *Tssk2*$^{-/-}$Line 1, and *Tssk2*$^{-/-}$ Line 2. Dashed lines represent deleted nucleotides, shown under the dashed lines.

A.

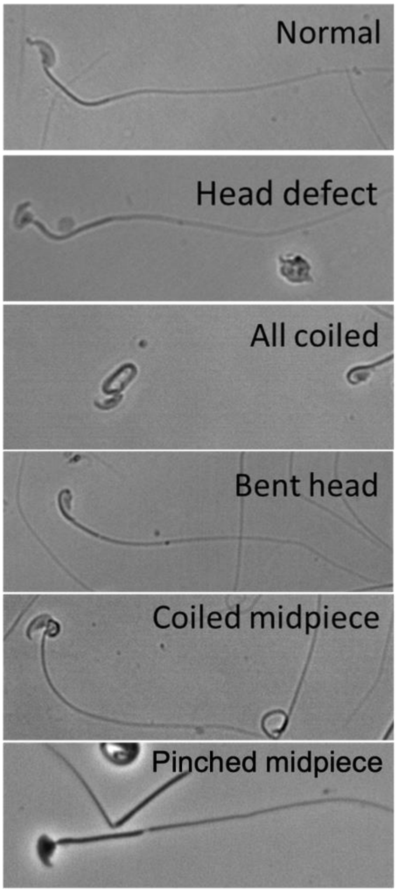

B.

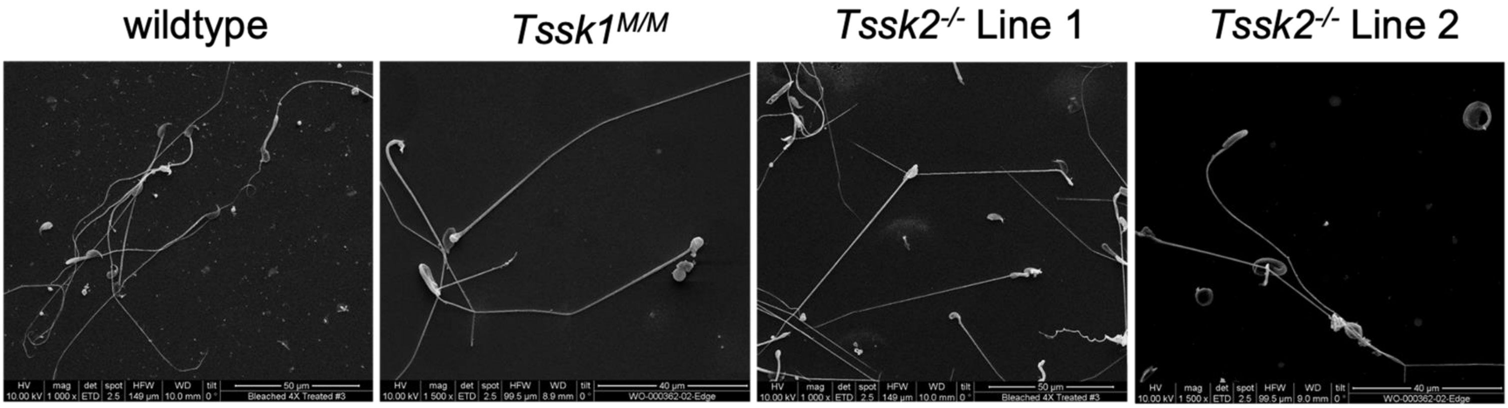

Supplement Figure S2. Abnormal sperm morphology images. A) Types of abnormal morphology observed in epididymal sperm from wildtype, *Tssk1*$^{M/M}$, *Tssk2*$^{-/-}$ Line 1, and *Tssk2*$^{-/-}$ Line 2 adult animals. **B)** Scanning electron microscopy images of sperm from wildtype, *Tssk1*$^{M/M}$, *Tssk2*$^{-/-}$ Line 1, and *Tssk2*$^{-/-}$Line 2 adult animals showing abnormal morphology. 1000x magnification.

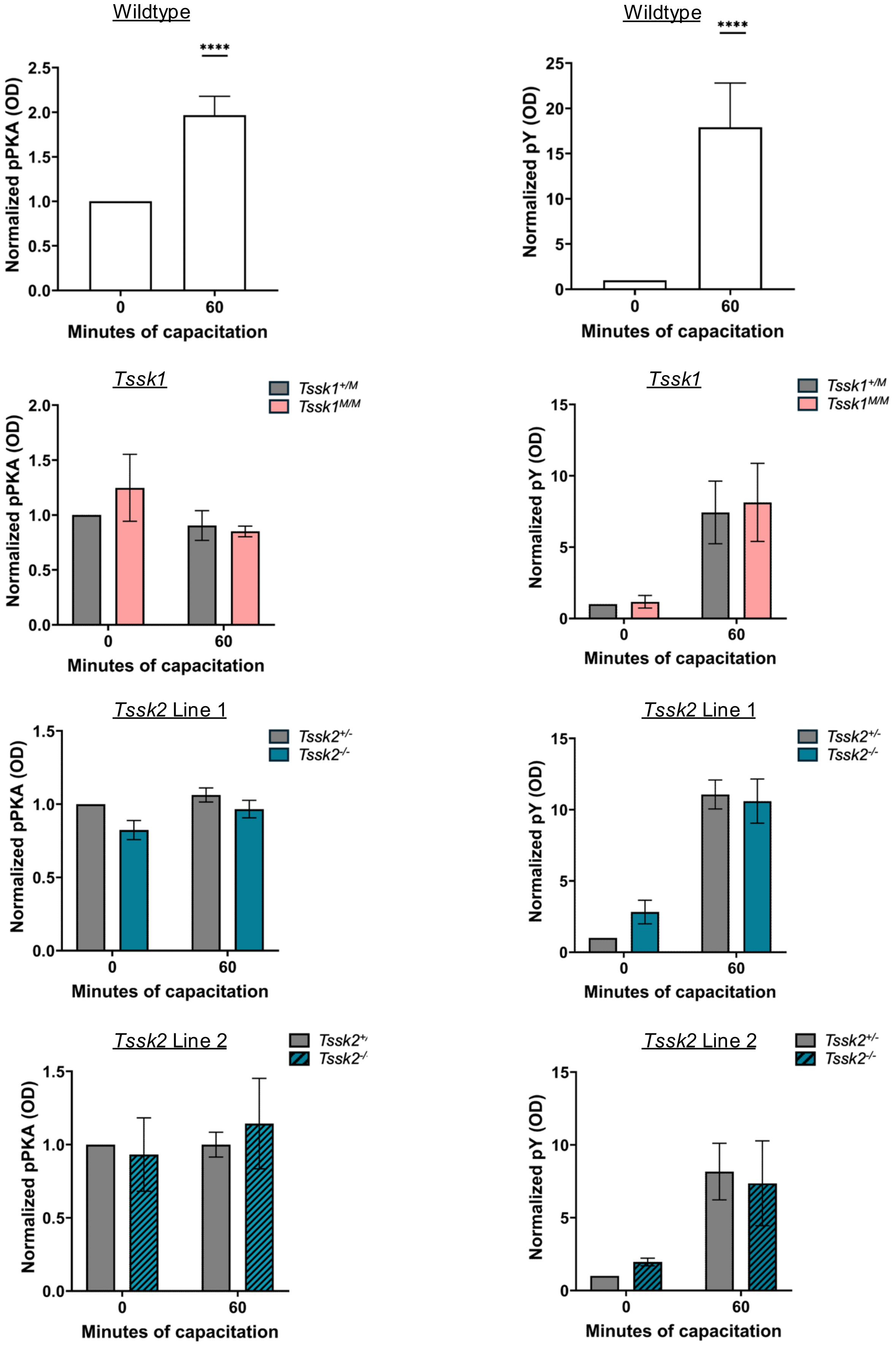

Supplement Figure S3. Quantitative analysis of optical density pPKAs and pY blots. A, B) Respective analysis of bands in the region marked between # in anti-pPKA blot and under the arrow in anti-pY blots in Figure 4C, normalized using tyrosine-hexokinase as control, indicated by arrow in anti-pY blots. Results express as mean + SEM as ****p<0.0001.

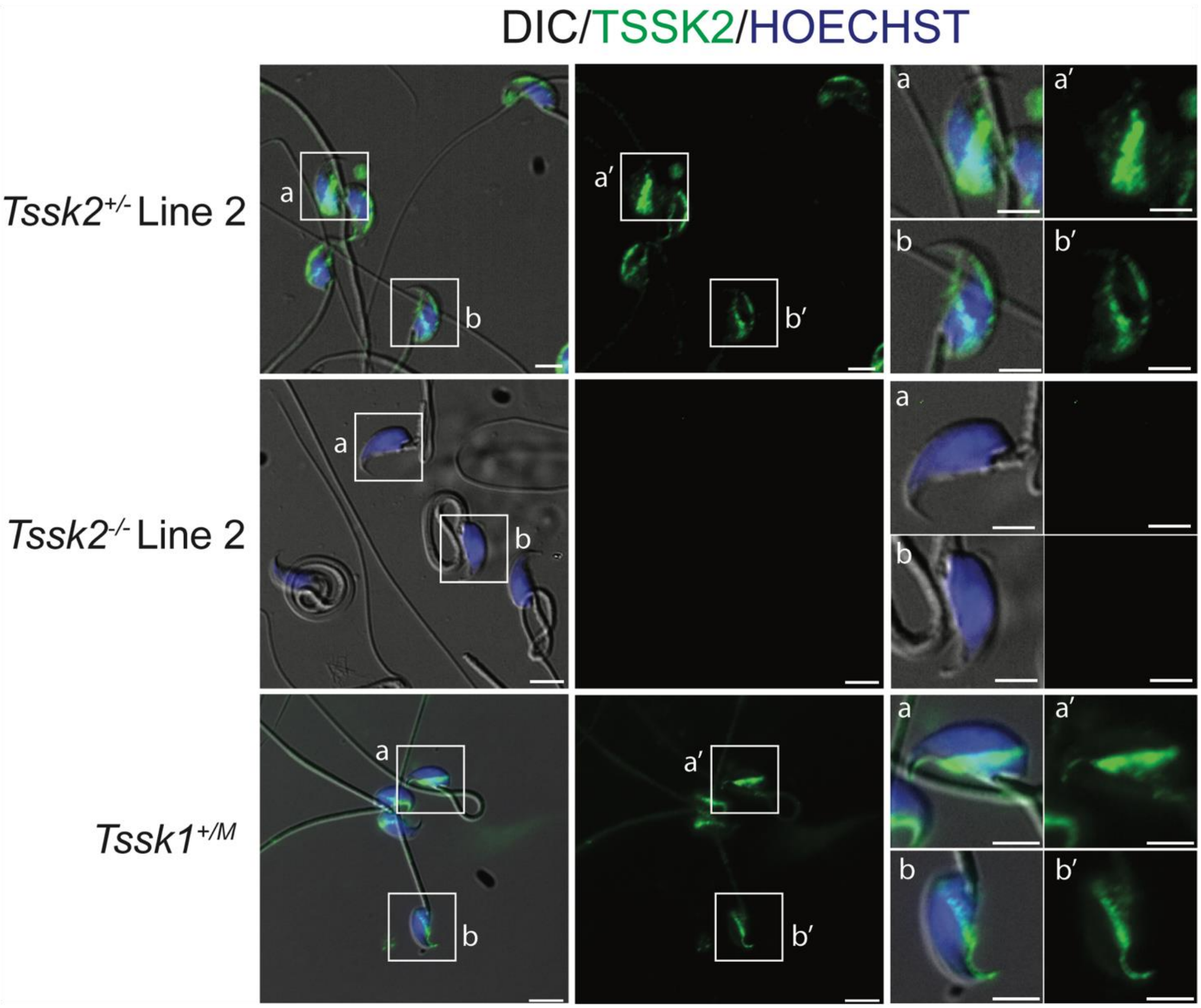

Supplement Figure S4. Additional immunofluorescence experiments using anti TSSK2 antibodies. Representative images show differential interference contrast (DIC) and epifluorescence microscopy of caudal sperm from *Tssk2*$^{+/-}$ Line 2, *Tssk2*$^{-/-}$ Line 2 and Tssk1$^{+/M}$. TSSK2 is shown in green and is absent in the TSSK2$^{-/-}$ sperm. Hoechst staining is represented in blue. Scale bar = 20 μm, insert scale bar = 5 μm.
